# Identification of somatic mutations in *EGFR*/*KRAS*/*ALK*-negative lung adenocarcinoma in never-smokers

**DOI:** 10.1186/gm535

**Published:** 2014-02-27

**Authors:** Jin Woo Ahn, Han Sang Kim, Jung-Ki Yoon, Hoon Jang, Soo Min Han, Sungho Eun, Hyo Sup Shim, Hyun-Jung Kim, Dae Joon Kim, Jin Gu Lee, Chang Young Lee, Mi Kyung Bae, Kyung Young Chung, Ji Ye Jung, Eun Young Kim, Se Kyu Kim, Joon Chang, Hye Ryun Kim, Joo Hang Kim, Min Goo Lee, Byoung Chul Cho, Ji Hyun Lee, Duhee Bang

**Affiliations:** 1 Department of Chemistry, Yonsei University, Seoul 120-752, Korea; 2 Department of Pharmacology, Pharmacogenomic Research Center for Membrane Transporters, Brain Korea 21 PLUS Project for Medical Sciences, Severance Biomedical Science Institute, Yonsei University College of Medicine, Seoul 120-752, Korea; 3 Yonsei Cancer Center, Division of Medical Oncology, Department of Internal Medicine, Yonsei University College of Medicine, Seoul 120-752, Korea; 4 College of Medicine, Seoul National University, Seoul 110-799, Korea; 5 Hwasung Public Health Center, Hwasung, Korea; 6 Yonsei University College of Medicine, Seoul 120-752, Korea; 7 Department of Pathology, Yonsei University College of Medicine, Seoul 120-752, Korea; 8 JE UK Institute for Cancer Research, Gumi City, Kyungbuk, Korea; 9 Department of Thoracic and Cardiovascular Surgery, Yonsei University College of Medicine, Seoul, Korea; 10 Division of Pulmonology, Department of Internal Medicine, Yonsei University College of Medicine, Seoul, Republic of Korea; 11 Department of Oral Biology, College of Dentistry, Yonsei University, Seoul, Republic of Korea

## Abstract

**Background:**

Lung adenocarcinoma is a highly heterogeneous disease with various etiologies, prognoses, and responses to therapy. Although genome-scale characterization of lung adenocarcinoma has been performed, a comprehensive somatic mutation analysis of *EGFR*/*KRAS*/*ALK*-negative lung adenocarcinoma in never-smokers has not been conducted.

**Methods:**

We analyzed whole exome sequencing data from 16 *EGFR*/*KRAS*/*ALK*-negative lung adenocarcinomas and additional 54 tumors in two expansion cohort sets. Candidate loci were validated by target capture and Sanger sequencing. Gene set analysis was performed using Ingenuity Pathway Analysis.

**Results:**

We identified 27 genes potentially implicated in the pathogenesis of lung adenocarcinoma. These included targetable genes involved in PI3K/mTOR signaling (*TSC1*, *PIK3CA*, *AKT2*) and receptor tyrosine kinase signaling (*ERBB4*) and genes not previously highlighted in lung adenocarcinomas, such as *SETD2* and *PBRM1* (chromatin remodeling), *CHEK2* and *CDC27* (cell cycle), *CUL3* and *SOD2* (oxidative stress), and *CSMD3* and *TFG* (immune response). In the expansion cohort (N = 70), *TP53* was the most frequently altered gene (11%), followed by *SETD2* (6%), *CSMD3* (6%), *ERBB2* (6%), and *CDH10* (4%). In pathway analysis, the majority of altered genes were involved in cell cycle/DNA repair (*P* <0.001) and cAMP-dependent protein kinase signaling (*P* <0.001).

**Conclusions:**

The genomic makeup of *EGFR*/*KRAS*/*ALK*-negative lung adenocarcinomas in never-smokers is remarkably diverse. Genes involved in cell cycle regulation/DNA repair are implicated in tumorigenesis and represent potential therapeutic targets.

## Background

Lung cancer is the leading cause of cancer deaths worldwide [1]. In 2008, 1.38 million deaths were attributed to lung cancer, accounting for approximately 20% of cancer-related deaths. Lung cancer is a highly heterogeneous disease with regard to its etiology, prognosis, and response to therapy, complicating both prevention and treatment [1]. Non-small cell lung cancer accounts for approximately 85% of newly diagnosed lung cancers and can be classified into two major histologic subtypes: adenocarcinoma (approximately 50% of cases) and squamous cell carcinoma (approximately 30%). Although the majority of lung cancer cases are attributed to tobacco smoke, approximately 25% of lung cancer cases worldwide occur in never-smokers. Lung cancers in never-smokers have distinct clinicopathologic characteristics and clinical outcomes [2,3].

The discovery of driver mutations, such as epidermal growth factor receptor (EGFR) and anaplastic lymphoma kinase (ALK), has led to remarkable improvements in personalized therapies for lung adenocarcinoma [4]. For example, erlotinib and gefitinib has been particularly efficacious in patients with EGFR mutations, and crizotinib in patients with ALK fusions [5,6]. Actionable genetic alterations that are treatable with therapeutic agents have been identified in approximately 50% of lung adenocarcinomas and include mutations in *EGFR*, *ERBB2*, *KRAS*, *ALK*, *BRAF*, *PIK3CA*, *AKT1*, *ROS1*, *NRAS*, and *MAP2K1*[4]. Therefore, identification of novel druggable targets in the remaining 50% of lung adenocarcinomas is a top research priority.

Comprehensive genomic approaches are being undertaken to accelerate the identification of new molecular targets and increase our understanding of the critical cellular and molecular mechanisms underlying lung cancer [7-13]. The first comprehensive mutation profiling of 623 genes in 188 adenocarcinomas identified 26 significantly mutated genes, including known oncogenes (*KRAS*, *EGFR*, *ERBB2*, *ERBB4*, *EPHA3* and other ephrin receptor genes, *KDR*, and *FGFR4*) and tumor suppressor genes (*TP53*, *CDKN2A*, *STK11*, *NF1*, *ATM*, *RB1*, and *APC*) [7]. Recent studies using next-generation sequencing identified new candidate driver mutations, including *RET1* rearrangements [8,14] and mutations in *CSMD3*[9], *MXRA5*[10], *U2AF1*, *RBM10*, and *ARID1A*[11], and *DACH1*, *CFTR*, *RELN*, *ABCB5*, and *HGF*[12]. Previous studies focused primarily on the identification of new driver genes according to mutation frequency and pattern; systematic pathway-based analysis has not been performed. Moreover, sample sizes in previous studies were insufficient to resolve rare driver mutations in lung adenocarcinoma.

In this study, we analyzed exome sequencing data from 16 *EGFR/KRAS/ALK*-negative tumors and paired normal samples and an applicable expansion cohort of 54 *EGFR*/*KRAS*-negative lung adenocarcinomas to identify novel somatic mutations in lung adenocarcinomas of never-smokers.

## Methods

### Preparation of clinical samples

Tumor and adjacent normal lung fresh tissues were obtained by surgical procedures. Clinical information including age, sex, smoking history, tumor histology, and tumor stage based on the seventh edition of the American Joint Committee on Cancer staging system was collected. Never-smokers were defined as patients who had a lifelong history of smoking fewer than 100 cigarettes. All patients provided informed consent. The study was approved by the Institutional Review Board of Severance Hospital (4-2011-0891) and conducted in accordance with the Helsinki Declaration [15].

*EGFR*/*KRAS* mutations were verified by Sanger sequencing and *ALK* rearrangement was detected by hybridization probes. To screen for *EGFR* and *KRAS* mutations by Sanger sequencing, we used the following primer sequences: 5′-CAGATGTTATCGAGGTCCGA-3′ and 5′-CAAGCAGAAGACGGCATACG-3′ to detect deletions in exon 19 of *EGFR*, 5′-CAAGCAGAAGACGGCATACG-3′ and 5′-GACCACCGAGATCTACACTC-3′ to detect the L858R mutation in exon 21 of *EGFR*, and 5′-GTGACATGTTCTAATATAGTCAC-3′ and 5′-TCTATTGTTGGATCATATTCGTC-3′ to detect mutations in codons 12 and 13 of *KRAS. ALK* rearrangements were verified using break-apart fluorescence *in situ* hybridization probes (Vysis LSI ALK Dual Color, Break Apart Rearrangement Probe; Abbott Molecular, Abbott Park, IL, USA). DNA was extracted from tissues using the DNeasy Blood & Tissue Kit (Qiagen, Valencia, CA, USA).

### Whole exome sequencing

Extracted DNA was sheared and a genomic library prepared using the NEBNext kit (New England BioLabs, Inc., Ipswich, MA, USA) according to the manufacturer’s instructions. Exon sequences were captured using the TruSeq Exome Enrichment Kit (Illumina, San Diego, CA, USA). Whole exome sequencing was performed using the Illumina HiSeq2000 platform. Sequencing data are accessible at Sequence Read Archive ([16] accession number [SRA:SRP022932]).

### Exome data analyses

The analysis flow chart is illustrated in Additional file 1: Figure S1. The references for software packages used for exome data analyses are summarized in Additional file 1. Briefly, all sequenced reads were aligned to the human reference genome National Center for Biotechnology Information build 37 (hg19) using Novoalign. Local re-aligning around indels and pair-end fixing was performed by GATK (version 1.4-21) and Picard, and PCR duplicates were removed using Picard. Quality scores were recalibrated by GATK.

Three variant calling programs were used to call single nucleotide variants: muTect (1.0.287783), VarScan (version 2.2.11), and GATK Unified Genotyper. Indels were called by the GATK Somatic Indel Detector with default parameters in paired sample mode.

Mutated loci were annotated using ANNOVAR and Polyphen2. Only non-synonymous single nucleotide variants and indels in coding exons and splicing sites were included. Known single nucleotide polymorphisms with minor allelic frequency >5% in the 1000 Genome Project Phase I East Asian (2012 April) and NHLBI Exome Sequencing Project 6500 (2012 Oct) were annotated and removed by ANNOVAR.

### Validation by molecular-inversion probe capture

The molecular-inversion probe (MIP) capture method was used to validate 1,401 candidate loci identified in whole exome sequencing. We designed 3,726 probes to capture the candidate loci (Additional file 2: Table S7). The microarray-based MIP preparation and capture experiment followed MIP standard-operating procedures with modifications in the preparation of probes (manuscript in preparation) [17].

For MIP probe hybridization, 1 μg of genomic DNA, 1.5 μl of Ampligase buffer (Epicentre, Madison, WI, USA), 1 μl of probe mixture (genomic DNA to probe ratio, 1:90), and distilled H_2_O were combined to give a total volume of 15 μl. The reaction was carried out for 5 minutes at 95°C and then the temperature was decreased to 60°C at a rate of 0.1°C per second followed by incubation for 24 hours at 60°C. After addition of 0.2 μl of Phusion polymerase (New England BioLabs Inc.), 1 μl of deoxyribonucleotide triphosphate (New England BioLabs, Inc.), 0.2 μl of Ampligase buffer (Epicentre), 4 units of Ampligase DNA ligase (Epicentre), and 0.3 μl of distilled H_2_O, the mixture was incubated for an additional 24 hours.

PCR products were purified with a Qiagen gel extraction kit, mixed equally based on concentrations determined using a Qubit 2.0 fluorometer (Invitrogen, Carlsbad, CA, USA), and sent for Illumina HiSeq2000 sequencing.

### Molecular-inversion probe capture data analysis

The raw data were aligned to the human reference genome (hg19) by Novoalign. Aligned data on the position of candidate loci were selected and transformed to pileup format by SAMtools. The candidate loci were defined as validated loci if the variant base was the same as that of whole exome sequencing and the following criteria were satisfied: variant allele frequency in tumor ≥5%, reads supporting variant allele in normal ≤2.

### Validation by Sanger sequencing

Candidate driver mutations and randomly selected validated loci were chosen for additional validation and appropriate primer pairs for Sanger sequencing were designed (Additional file 2: Table S8). PCR products were purified and sent for Sanger sequencing (Macrogen, Seoul, Korea). Sequencing data were analyzed with SeqMan (DNASTAR, Madison, WI, USA).

### Canonical pathway analysis

The pathway analysis was performed through the use of Ingenuity Pathway Analysis (Ingenuity® Systems [18]). Pathways associated with a set of focus genes were identified from the Ingenuity Pathways Analysis library of canonical pathways. The *P*-value was measured to decide the likelihood that the association between focus genes and a given pathway was due to random chance. The more focus genes involved, the more likely the association is not due to random chance, and thus the more significant the *P*-value. A right-tailed Fisher’s exact test was used to calculate a *P*-value determining the probability of an association between the focus genes and the canonical pathway.

### Statistical analyses

Continuous clinical data (for example, age) were compared using independent Student’s *t*-tests. Categorical data (for example, sex, ethnicity, stage, mutation frequency) were compared using a chi-squared test. The false-discovery rate was corrected for multiple comparisons using the method of Benjamini and Hochberg. All statistical tests were two-tailed, and a *P*-value ≤0.05 was considered statistically significant. Data analyses were performed using R statistical software version 2.15.3 [19].

## Results

### Exome sequencing of *EGFR*/*KRAS*/*ALK*-negative tumors in never-smokers

We screened 230 surgically resected lung adenocarcinoma samples to identify *EGFR*/*KRAS*/*ALK*-negative tumors in never-smokers. A total of 16 tumors (7% of all non-small cell lung cancers) were eligible for exome sequencing. Tumor and normal samples had an average sequencing depth of 51.9× and 52.0× respectively with average coverage of 94.6% for each (Additional file 1: Table S1). Somatic variants were validated by target-capture sequencing (154-fold depth; average coverage of 94%) and Sanger sequencing, which had a concordance rate of 94% with exome sequencing (Additional file 1: Tables S2 and S3). We detected a median number of 10 non-synonymous mutations and indels per tumor (range 3 to 27; Additional file 1: Table S4). The median rate of non-synonymous mutation was 0.32 mutations per megabase, which was comparable to that in previous reports for never-smokers [11,12]. The average ratio of transitions to transversions was 1.95; G:C → A:T transitions (37%) were the most frequent followed by A:T → G:C transitions (21%), consistent with a previous lung cancer exome study [11]. Validated loci were further analyzed for functional prediction of amino acid changes using four different prediction algorithms (SIFT, Polyphen2, LRT, and Mutation Taster) (Additional file 1).

**Figure 1:**
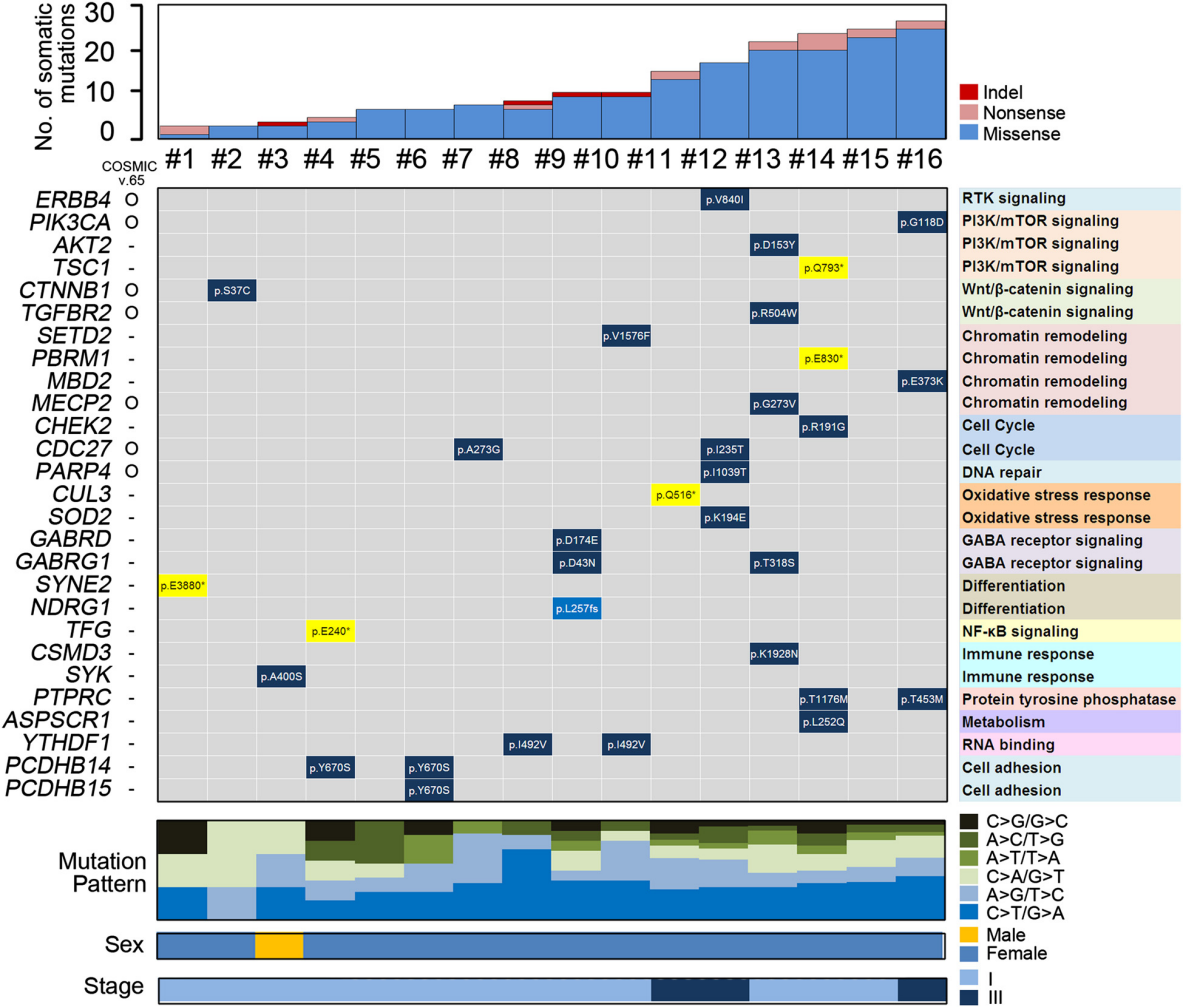
Mutation summary of 16 EGFR/KRAS/ALK-negative lung adenocarcinomas.

Non-synonymous somatic mutations in 16 *EGFR*/*KRAS*/*ALK*-negative tumors are summarized in Figure 1 (full mutation information is provided in Additional file 1: Table S5). Overall, 14 of 16 patients (87%) had a putative non-synonymous mutation. Although *EGFR*/*KRAS*/*ALK*-negative tumors harbored heterogeneous mutation profiles, somatically altered genes were functionally classified as follows: major mitogenic and targetable pathways such as PI3K/mTOR signaling (*TSC1*, *PIK3CA*, *AKT2*), receptor tyrosine kinase signaling (*ERBB4*), protein tyrosine phosphatase (*PTPRC*), cell cycle (*CHEK2*, *CDC27*), and DNA repair (*PARP4*); tumor suppressor pathways including chromatin remodeling (*SETD2*, *PBRM1*, *MBD2*, *MECP2*), Wnt signaling (*CTNNB1*, *TGFBR2*), and NF-κB signaling (*TFG*); oxidative stress response (*CUL3*, *SOD2*) and differentiation (*SYNE2*, *NDRG1*) (similar to lung squamous cell carcinoma [20]); pathways not previously highlighted in carcinogenesis such as gamma-aminobutyric acid receptor signaling (*GABRD*, *GABRG1*) and immune response (*CSMD3*, *SYK*); as well as *YTHDF1* (suggested role in RNA binding) and *PCDHB14* (role in cell adhesion).

### Investigation of functional domains in altered genes

Of 32 loci shown in Figure 1, seven were found in the Catalogue of Somatic Mutations in Cancer (COSMIC v.65): *ERBB4 V840I*, *PIK3CA G118D*, *CTNNB1 S37C*, *TGFBR2 R504W*, *MECP2 G273V*, *CDC27 A273G*, and *PARP4 I1039T*. In addition, *PIK3CA G118D* (in squamous cell carcinoma), *CTNNB1 S37C* (in adenocarcinoma), and *MECP2 G273V* (in adenocarcinoma) were previously reported in cases of lung cancer.

To explore the functional effects of somatic variants, we investigated functional domains in altered loci. Seventeen loci of 27 genes shown in Figure 1 were located in functional domains, including kinase domains (*ERBB4* (tyrosine kinase domain), *AKT2* (serine/threonine kinase domain), *TGFBR2* (tyrosine kinase domain), *SYK* (protein kinase domain)) or domains involved in oncogenic kinase activation (*TSC1* (TPR/MLP1/MLP2-like protein)); in histone modification domains (*SETD2* (SET domain), *PBRM1* (bromo domain), *MBD2* (methyl CpG binding domain), *MECP2* (methyl CpG binding domain)); in oxidative stress response/differentiation domains (*CUL3* (cullin domain), *SOD2* (superoxide dismutase domain), *NDRG1* (Ndr family domain)); in ion-channel domains (*GABRD* (ion-channel binding domain), *GABRG1* (ion-channel transmembrane domain)); or were for cell cycle checkpoint proteins (*CHEK2* (fork head associated domain)).

### Somatic mutations in an expansion cohort of *EGFR*/*KRAS*-negative tumors in never-smokers

To validate and expand our mutation analysis in lung adenocarcinoma in never-smokers, we collected an expansion dataset from five available lung adenocarcinoma studies [9-13] and a The Cancer Genome Atlas lung carcinoma study [21], with no overlap with the study of Imielinski *et al*. [11]. Clinical information of all patients including sex, age, tumor stage, and ethnicity is given in Table 1. A total of 54 *EGFR/KRAS*-negative tumors from never-smokers were analyzed. Information on non-synonymous and splicing site mutations were extracted from a pooled dataset. The median rate of non-synonymous mutations in *EGFR/KRAS*-negative never-smokers was approximately 0.65 mutations per megabase and the median number of non-synonymous mutations per patient was 19.0. The average ratio of transitions to transversions was 1.07 and G:C → A:T transitions (40%) were the most frequent, consistent with our data.

**Table 1:** Patient characteristics (N = 70)

**Characteristic**	**This study**	**Expansion cohort**^**a**^	**TCGA-LUAD**^**a**^
Number of patients	16	40	14
Age (years)			
Median	58.5	63	69
Range	47 to 79	36 to 87	52 to 79
Sex (%)			
Male	1 (6)	7 (17)	2 (14)
Female	15 (94)	29 (73)	12 (86)
n/a	0	4 (10)	0
Clinical stage (%)			
I	13 (81)	24 (60)	9 (65)
II	0	6 (15)	2 (14)
III	3 (19)	6 (15)	3 (21)
IV	0	1 (2)	0
n/a	0	3 (8)	0
Ethnic group (%)			
Caucasian	0	16 (40)	14 (100)
Asian	16 (100)	24 (60)	0

^a^Never-smokers with *EGFR*/*KRAS*-negative adenocarcinoma. Mb, megabase; n/a, not available; SD, standard deviation; TCGA-LUAD, The Cancer Genome Atlas - Lung adenocarcinoma.

**Figure 2:**
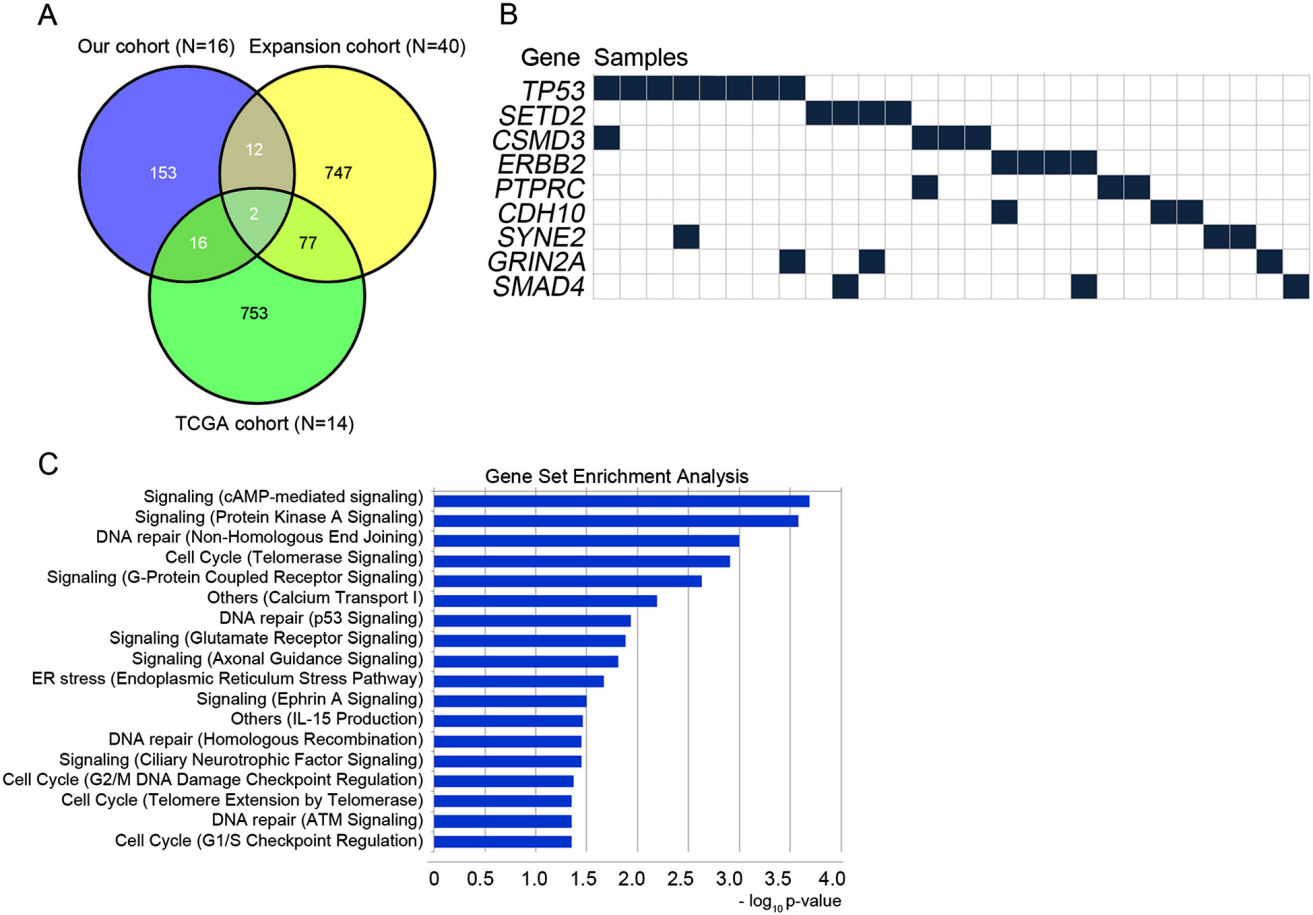
**Distribution and pathway analysis of somatic mutations. (A)** Venn diagram plot comparing somatically altered genes among our cohort (N = 16), the expansion cohort (N = 40), and TCGA-LUAD cohort (N = 14). **(B)** Gene profiles across *EGFR*/*KRAS*-negative tumors from never-smokers. **(C)** Pathway analysis of altered genes in *EGFR*/*KRAS*-negative lung adenocarcinoma from never smokers (N = 70). The most significant functions are shown. TCGA-LUAD, The Cancer Genome Atlas - Lung adenocarcinoma.

Comparison of altered genes among the three cohorts is shown in Figure 2. *SETD2* and *CSMD3* were altered in all three cohorts (Figure 2A). Commonly altered genes with information on affected loci, amino acid changes, and functional predictions are summarized in Table 2 (full information is provided in Additional file 1: Table S6). The most frequently mutated gene was *TP53*, which was altered in 11% of tumors, followed by *SETD2* (6%, 4 of 70 cases), *CSMD3* (6%, 4 of 70 cases), and *ERBB2* (6%, 4 of 70 cases). *PTPRC*, *SYNE2*, *GRIN2A*, *CDH10*, and *SMAD4* were each altered in 3 of 70 cases (4%). SETD2 interacts with p53 and regulates genes downstream of p53 in addition to increasing p53 stability [22]. Mutations in *SETD2* were nonsense mutations in three cases and missense mutation in one case. The missense mutation V1576F is located in the SET domain; one nonsense mutation, R839*, is a truncating mutation upstream of the SET domain, and two nonsense mutations, Q1981* and K2067*, are truncating mutation upstream of the WW domain. In addition to known cancer driver genes such as *ERBB2* (6% of cases), *NRAS* (3%), *MET* (3%), *PIK3CA* (1%), *AKT2* (1%), *TSC1* (1%), and *ERBB4* (1%), several putative cancer genes were identified, such as *PTPRC*[23], *SYNE2*[24], *GRIN2A*[25], and *CDH10*[26]. The mutation pattern is summarized in Figure 2B.

**Table 2:** **Mutated genes and loci information for *EGFR*/ *KRAS *-negative lung adenocarcinomas from our cohort, the expansion cohort, and The Cancer Genome Atlas cohort**

**Gene**	**Mutation frequency in our set (N = 16)**	**Mutation frequency in expansion set (N = 40)**	**Mutation frequency in TCGA set (N = 14)**	**Total (N)**	**AA substitution**	**COSMIC v.65**	**Number of damage algorithms**
*TP53*	-	6 of 40	2 of 14	8 of 70	K93R	O	4 of 4
					122_122del	O	n/a
					Y124C	O	4 of 4
					C203F	O	4 of 4
					G206S	O	4 of 4
					R209W	O	4 of 4
					E219*	O	2 of 4
					G295A	O	4 of 4
*SETD2*	1 of 16	2 of 40	1 of 14	4 of 70	R839*	-	1 of 2
					V1576F	-	3 of 3
					Q1981*	-	2 of 2
					K2067*	-	0 of 1
*CSMD3*	1 of 16	1 of 40	2 of 14	4 of 70	P667S	O	3 of 4
					M1440I	-	3 of 4
					K1928N	-	3 of 4
					Y2028C	-	3 of 4
*PTPRC*	2 of 16	1 of 40	-	3 of 70	Y444N	O	3 of 4
					T453M	-	3 of 4
					T1176M	-	4 of 4
*SYNE2*	1 of 16	2 of 40	-	3 of 70	2579_2580del	-	n/a
					E3880*	-	n/a
					E3903K	O	3 of 4
*GRIN2A*	-	3 of 40	-	3 of 70	L307Q	-	3 of 4
					N886S	O	0 of 4
					T1069M	O	3 of 4
*CDH10*	-	2 of 40	1 of 14	3 of 70	E171K	O	4 of 4
					D315H	O	1 of 4
					R472C	O	4 of 4
*ERBB2*	-	-	3 of 14	3 of 70	E740delinsEAYVM (2)	-	n/a
					G746delinsVC	O	n/a
*SMAD4*	-	-	3 of 14	3 of 70	S242*	-	1 of 2
					R361S	O	4 of 4
					D493N	O	3 of 4
*CDC27*	2 of 16	-	-	2 of 70	I235T	-	4 of 4
					A273G	O	3 of 4
*GABRG1*	2 of 16	-	-	2 of 70	D43N	-	2 of 4
					T318S	-	3 of 4
*YTHDF1*	2 of 16	-	-	2 of 70	I492V (2)	-	4 of 4
*PCDHB14*	2 of 16	-	-	2 of 70	Y670S (2)	-	2 of 3
*MECP2*	1 of 16	-	1 of 14	2 of 70	R168L	-	2 of 3
					G273V	O	3 of 3
*NRAS*	-	2 of 40	-	2 of 70	Q61H	O	2 of 4
					Q61K	O	3 of 4
*MET*	-	-	2 of 14	2 of 70	Y1021*	-	2 of 2
					1027_1028del	-	NA
*EPHA2*	-	2 of 40	-	2 of 70	P147L	O	4 of 4
					P278S	O	2 of 4
*CHD2*	-	2 of 40	-	2 of 70	S391fs	-	n/a
					splicing(c.5153)	O	n/a
*MST1*	-	-	2 of 14	2 of 70	406_407del	-	n/a
					R535G	-	3 of 4
*ERBB4*	1 of 16	-	-	1 of 70	V840I	O	4 of 4
*PIK3CA*	1 of 16	-	-	1 of 70	G118D	O	2 of 3
*AKT2*	1 of 16	-	-	1 of 70	D153Y	-	4 of 4
*TSC1*	1 of 16	-	-	1 of 70	Q793*	-	2 of 2
*CTNNB1*	1 of 16	-	-	1 of 70	S37C	O	4 of 4
*TGFBR2*	1 of 16	-	-	1 of 70	R504W	O	4 of 4
*PBRM1*	1 of 16	-	-	1 of 70	E830*	-	2 of 2
*MBD2*	1 of 16	-	-	1 of 70	E373K	-	4 of 4
*CHEK2*	1 of 16	-	-	1 of 70	R191G	-	3 of 4
*PARP4*	1 of 16	-	-	1 of 70	I1039T	O	4 of 4
*CUL3*	1 of 16	-	-	1 of 70	Q516*	-	2 of 2
*SOD2*	1 of 16	-	-	1 of 70	K194E	-	3 of 4
*GABRD*	1 of 16	-	-	1 of 70	D174E	-	4 of 4
*NDRG1*	1 of 16	-	-	1 of 70	L257fs	-	n/a
*TFG*	1 of 16	-	-	1 of 70	E240*	-	1 of 1
*SYK*	1 of 16	-	-	1 of 70	A400S	-	4 of 4
*ASPSCR1*	1 of 16	-	-	1 of 70	L252Q	-	3 of 3
*PCDHB15*	1 of 16	-	-	1 of 70	Y670S	-	3 of 3

COSMIC, Catalogue of Somatic Mutations in Cancer; n/a, not available; TCGA, The Cancer Genome Atlas.

Pathway analysis of 1,760 genes that were altered in 70 *EGFR*/*KRAS*-negative tumors of never-smokers revealed alterations in genes related to DNA repair and the cell cycle, including components of p53/ATM signaling, G1/S or G2/M checkpoint regulation, and non-homologous end joining (Figure 2C). The most significantly enriched pathway was cAMP-dependent protein kinase A signaling, which can activate the mitogen-activated protein kinase cascade in lung adenocarcinoma [27]. Other enriched functions of altered genes were calcium transport (*P* = 0.006), axonal guidance (*P* = 0.015), and Ephrin A signaling (*P* = 0.031).

## Discussion

**Figure 3:**
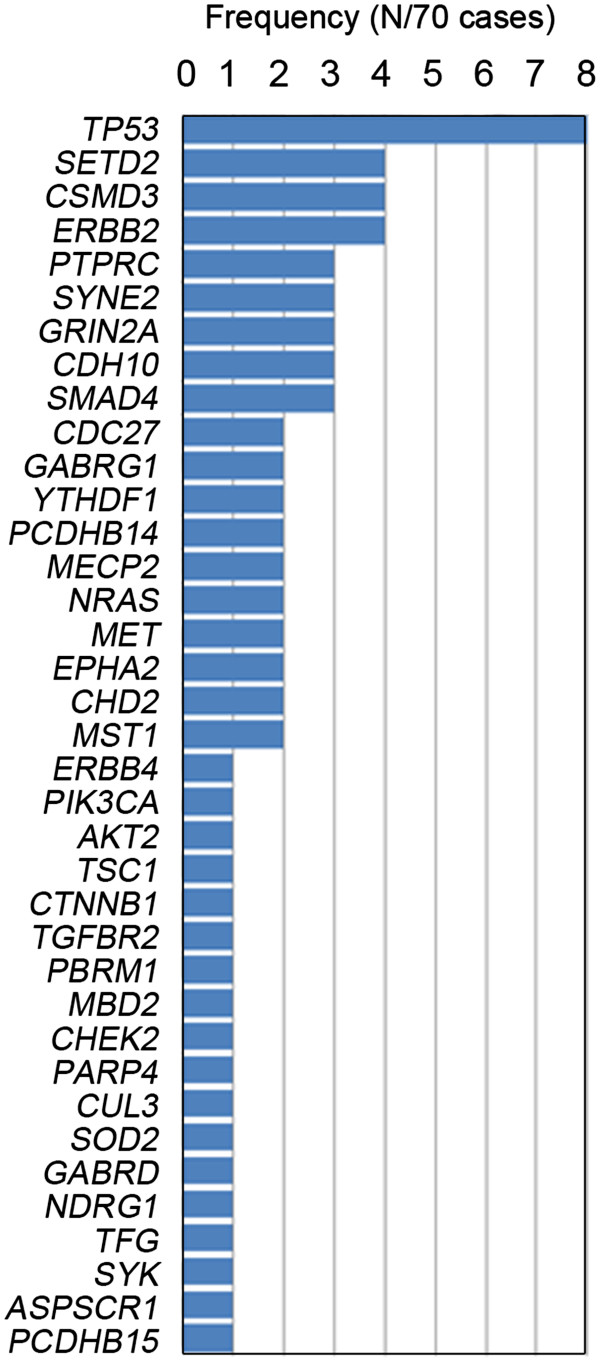
**Significantly mutated genes in *EGFR*/*KRAS*-negative lung adenocarcinomas from never-smokers (N = 70).**

The somatic mutation profile in lung adenocarcinomas lacking targetable *EGFR* or *KRAS* mutations or *ALK* rearrangements in never-smokers is highly complex. Our exome analysis of 70 tumors identified several common mutations involving the known cancer genes *TP53*, *NRAS*, *ERBB2*, *PIK3CA*, and *CTNNB1*, but also mutations in *SETD2*, *CSMD3*, *PTPRC*, and *SYNE2* (Figure 3)*.*

SETD2 (mutated in 6% of cases) is a histone methyltransferase that is involved in transcriptional elongation and chromatin remodeling. Interaction with p53 is facilitated by the SET and WW domains and might increase p53 stability [22]. Interestingly, *SETD2* and *TP53* mutations were mutually exclusive in lung adenocarcinoma of never-smokers (Figure 2B). CSMD3 (mutated in 6% of cases) is a transmembrane protein with CUB and sushi multiple domains that is thought to function in protein-protein interactions and the immune response. Recent studies showed that loss of CSMD3 increases proliferation of airway epithelial cells [9] and may be involved in tumorigenesis in lung cancer. In our study, missense mutations (P667S, M1440I, K1928N, and Y2028C) in *CSMD3* were predicted to be deleterious to protein function. PTPRC (mutated in 4% of cases) is a member of the protein tyrosine phosphatase family and regulates a variety of cellular processes including cell growth, differentiation, and tumorigenesis. PTPRC regulates the JAK/STAT signaling pathway and functional defects can activate JAK/STAT signaling [23]. We observed three missense mutations (Y444N, T453M, T1176M) in *PTPRC*, all of which were predicted to be deleterious. SYNE2 plays a role in cadherin-mediated cell-cell adhesion and regulates the Wnt signaling pathway [24].

We identified several targetable pathways in *EGFR*/*KRAS*/*ALK*-negative lung adenocarcinoma including PI3K/mTOR signaling (*TSC1*, *PIK3CA*, *AKT2*), receptor tyrosine kinase signaling (*ERBB4*), cell cycle regulation (*CHEK2*, *CDC27*), and DNA repair (*PARP4*). PI3K pathway inhibitors and cell cycle inhibitors are actively under investigation for lung adenocarcinoma in preclinical and early phase clinical trials [28,29]. A current mutation screening program for tailored targeted therapies is also on-going in 1,000 patients with advanced lung adenocarcinoma based on 10 single driver mutations: *KRAS* (25%), *EGFR* (23%), *ALK* rearrangements (6%), *BRAF* (3%), *PIK3CA* (3%), *MET* amplifications (2%), *ERBB2* (1%), *MEK1* (0.4%), *NRAS* (0.2%), and *AKT1* (<0.1%) [30]. Additional genomic alterations will be incorporated in a comprehensive manner based on next-generation sequencing data.

More than 200 putative cancer-causing genes have been identified in recent genomic landscape studies using next-generation sequencing technology, and several cellular processes not previously implicated in cancer have been revealed, such as chromatin remodeling, splicing, and ubiquitination [31,32]. We identified alterations in genes involved in chromatin remodeling (*PBRM1*, *SETD2*), oxidative stress (*CUL3*, *SOD2*), immune response (*CSMD3*, *SYK*), and gamma-aminobutyric acid receptor signaling (*GABRD*, *GABRG1*) in lung adenocarcinoma. Interestingly, although somatic mutation is rare in *EGFR*/*KRAS*/*ALK*-negative lung adenocarcinoma of never-smokers, the *PCDHB14* (cell adhesion) Y670S mutation and *YTHDF1* (RNA binding) I492V mutations were each found in two cases (12.5%). Future studies to elucidate the role of these newly implicated functions in tumorigenesis are warranted.

## Conclusions

We identified novel somatic mutations in *EGFR*/*KRAS*/*ALK*-negative lung adenocarcinoma in never-smokers and investigated the mutation frequency of altered genes. *EGFR*/*KRAS*/*ALK*-negative lung adenocarcinoma in never-smokers is highly heterogeneous at the somatic mutation level. However, most of the altered genes were involved in the cell cycle, and might represent novel therapeutic targets in lung adenocarcinoma. Future research on the functional role of chromatin remodeling, oxidative stress/differentiation, and the immune response will enhance our understanding of the mechanisms of tumorigenesis.

## Abbreviations

ALK: anaplastic lymphoma kinase; COSMIC: Catalogue of Somatic Mutations in Cancer; EGFR: epidermal growth factor receptor; MIP: molecular-inversion probe; PCR: polymerase chain reaction; TCGA: The Cancer Genome Atlas.

## Competing interests

The authors declare that they have no competing interests.

## Authors’ contributions

JWA and HSK analyzed the data and wrote the manuscript. JKY, HJ, SE, and SMH performed the experiments and designed the molecular inversion probes for validation. HSS performed pathologic review. HJK, DJK, JGL, CYL, MKB, and KYC prepared the samples for exome sequencing. JYJ, EYK, SKK, JC, and MGL helped to revise the manuscript. HRK and JHK provided clinical information. BCC, JHL, and DB designed and managed the study. All authors read and approved the final manuscript.

## Supplementary Material

Additional file 1: Figure S1 Analysis flow chart for exome-sequencing data. **Table S1.** Summary of depth and coverage of whole exome sequencing. **Table S2.** Summary of depth and coverage in target capture sequencing for validation. **Table S3.** Validation results using target capture sequencing and Sanger sequencing. **Table S4.** Summary of validated somatic exonic mutations in *EGFR*/*KRAS*/*ALK*-negative lung adenocarcinomas. **Table S5.** Somatic mutations in *EGFR*/*KRAS*/*ALK*-negative lung adenocarcinoma exomes. **Table S6.** Mutated genes and loci information in *EGFR*/*KRAS*-negative lung adenocarcinoma. Click here for file

Additional file 2: Table S7 Sequences of molecular inversion probes. **Table S8.** Sequences of primers used for Sanger sequencing. Click here for file
