# Supplementary material for: Identification of somatic mutations in EGFR/KRAS/ALK-negative lung adenocarcinoma in never-smokers
Source: Genome Med. 2014 Feb 27;6(2):18. doi: 10.1186/gm535 (PMC3979047; doi:10.1186/gm535)
Supplement: Additional file 1: Figure S1 — Analysis flow chart for exome-sequencing data. Table S1. Summary of depth and coverage of whole exome sequencing. Table S2. Summary of depth and coverage in target capture sequencing for validation. Table S3. Validation results using target capture sequencing and Sanger sequencing. Table S4. Summary of validated somatic exonic mutations in EGFR/KRAS/ALK-negative lung adenocarcinomas. Table S5. Somatic mutations in EGFR/KRAS/ALK-negative lung adenocarcinoma exomes. Table S6. Mutated genes and loci information in EGFR/KRAS-negative lung adenocarcinoma. [file gm535-S1.doc]

**Supplemental Information**

**Identification of Somatic Mutations in *EGFR*/*KRAS*/*ALK*-negative Lung Adenocarcinoma in Never-Smokers**

Jin Woo Ahn,^1^* Han Sang Kim,^2,3^* Jung-Ki Yoon,^4,5^* Hoon Jang,^1^ Soo Min Han,^2^ Sungho Eun,^6^ Hyo Sup Shim,^7^ Hyun-Jung Kim,^8^ Dae Joon Kim,^9^ Jin Gu Lee,^9^ Chang Young Lee,^9^ Mi Kyung Bae,^9^ Kyung Young Chung,^9^ Ji Ye Jung,^10^ Eun Young Kim,^10^ Se Kyu Kim,^10^ Joon Chang,^10^ Hye Ryun Kim,^3^ Joo Hang Kim,^3^ Min Goo Lee,^2^ Byoung Chul Cho,^3$^ Ji Hyun Lee,^11$^ Duhee Bang^1$^

**Authors’ Affiliations**

^1^Department of Chemistry, Yonsei University, Seoul 120-752, Korea

^2^Department of Pharmacology, Pharmacogenomic Research Center for Membrane Transporters, Brain Korea 21 PLUS Project for Medical Sciences, Severance Biomedical Science Institute, Yonsei University College of Medicine, Seoul 120-752, Korea

^3^Yonsei Cancer Center, Division of Medical Oncology, Department of internal Medicine, Yonsei University College of Medicine, Seoul 120-752, Korea

^4^College of Medicine, Seoul National University, Seoul 110-799, Korea

^5^Hwasung Public Health Center, Hwasung, Korea

^6^College of Medicine, Yonsei University, Seoul 120-752, Korea

^7^Department of Pathology, Yonsei University College of Medicine, Seoul 120-752, Korea

^8^JE UK Institute for Cancer Research, Gumi City, Kyungbuk, Korea

^9^Department of Thoracic and Cardiovascular Surgery, Yonsei University College of Medicine, Seoul, Korea

^10^Division of Pulmonology and Critical Care Medicine, Department of Internal Medicine, Yonsei University College of Medicine, Seoul, Republic of Korea

^11^Department of Oral Biology, College of Dentistry, Yonsei University, Seoul, Republic of Korea

**Table of Contents**

**1. Supplemental Figures**

Figure S1. Analysis flow chart for exome sequencing data 3

**2.Supplemental Tables**

Table S1. Summary of depth and coverage of whole exome sequencing 4

Table S2. Summary of depth and coverage in target capture sequencing for validation 5

Table S3. Validation results using target capture sequencing and Sanger sequencing 6

Table S4. Summary of validated somatic exonic mutations in *EGFR*/*KRAS*/*ALK*-negative lung adenocarcinomas 9

Table S5. Somatic mutations in *EGFR*/*KRAS*/*ALK*-negative lung adenocarcinoma exomes 10

Table S6. Mutated genes and loci information in *EGFR*/*KRAS*-negative lung adenocarcinoma 17

Table S7. Sequences of molecular inversion probes (MIPs) 20

Table S8. Sequences of primers used for Sanger sequencing 21

**3. References for exome analysis** 22

**Figure S1.** Analysis flow chart for exome sequencing data

**Table S1.** Summary of depth and coverage of whole exome sequencing

| Sample | | Depth of Coverage | 1× (%) | 5× (%) | 10× (%) | 15× (%) | 20× (%) |
| --- | --- | --- | --- | --- | --- | --- | --- |
| TNA#1 | Tumor | 54.81 | 94.52 | 91.17 | 87.87 | 84.29 | 80.37 |
|  | Normal | 47.64 | 94.39 | 90.56 | 86.56 | 82.18 | 77.36 |
| TNA#2 | Tumor | 76.91 | 93.81 | 91.27 | 89.46 | 87.74 | 85.91 |
|  | Normal | 61.43 | 93.82 | 90.79 | 88.35 | 85.90 | 83.17 |
| TNA#3 | Tumor | 36.83 | 92.95 | 88.41 | 83.33 | 77.38 | 70.54 |
|  | Normal | 49.41 | 93.17 | 89.56 | 86.25 | 82.74 | 78.69 |
| TNA#4 | Tumor | 64.25 | 92.68 | 89.16 | 86.05 | 82.83 | 79.35 |
|  | Normal | 58.71 | 93.46 | 89.97 | 86.59 | 82.94 | 78.95 |
| TNA#5 | Tumor | 36.83 | 94.55 | 89.37 | 75.95 | 68.85 | 54.91 |
|  | Normal | 59.14 | 94.68 | 91.74 | 85.36 | 81.78 | 73.87 |
| TNA#6 | Tumor | 72.82 | 94.95 | 91.62 | 84.84 | 81.36 | 74.18 |
|  | Normal | 42.17 | 94.15 | 89.11 | 77.08 | 70.92 | 59.10 |
| TNA#7 | Tumor | 37.60 | 94.42 | 89.40 | 76.69 | 69.93 | 56.43 |
|  | Normal | 30.70 | 94.36 | 88.61 | 72.48 | 63.68 | 46.23 |
| TNA#8 | Tumor | 56.04 | 96.56 | 92.86 | 88.94 | 84.89 | 80.51 |
|  | Normal | 56.00 | 96.66 | 92.99 | 89.11 | 85.14 | 80.84 |
| TNA#9 | Tumor | 44.35 | 95.27 | 91.79 | 87.72 | 83.16 | 77.97 |
|  | Normal | 43.60 | 95.10 | 91.66 | 87.28 | 82.08 | 75.97 |
| TNA#10 | Tumor | 38.03 | 95.93 | 91.03 | 85.26 | 78.71 | 71.31 |
|  | Normal | 53.84 | 96.60 | 93.22 | 89.56 | 85.72 | 81.41 |
| TNA#11 | Tumor | 41.50 | 93.26 | 88.94 | 84.32 | 79.17 | 73.43 |
|  | Normal | 42.81 | 92.53 | 87.82 | 82.76 | 77.17 | 71.12 |
| TNA#12 | Tumor | 62.80 | 95.02 | 92.15 | 88.91 | 85.42 | 81.66 |
|  | Normal | 75.04 | 95.40 | 92.75 | 89.95 | 87.05 | 84.03 |
| TNA#13 | Tumor | 42.67 | 93.77 | 89.95 | 85.32 | 79.99 | 74.06 |
|  | Normal | 43.45 | 93.89 | 90.28 | 85.83 | 80.59 | 74.64 |
| TNA#14 | Tumor | 61.29 | 95.11 | 92.00 | 88.58 | 84.93 | 81.00 |
|  | Normal | 62.28 | 94.92 | 92.30 | 89.37 | 86.03 | 82.25 |
| TNA#15 | Tumor | 49.61 | 94.82 | 90.90 | 81.75 | 76.81 | 66.45 |
|  | Normal | 41.63 | 94.69 | 90.24 | 78.67 | 72.40 | 59.81 |
| TNA#16 | Tumor | 54.89 | 95.44 | 92.95 | 85.88 | 81.17 | 70.20 |
|  | Normal | 64.16 | 95.49 | 93.44 | 88.84 | 85.93 | 78.70 |

**Table S2.** Summary of depth and coverage in target capture sequencing for validation

| Sample | Fold death of coverage | 1× (%) | 5× (%) | 10× (%) | 15× (%) | 20× (%) |
| --- | --- | --- | --- | --- | --- | --- |
| TNA#1-T | 398.70 | 97.92 | 97.10 | 96.55 | 95.80 | 95.19 |
| TNA#1-N | 190.14 | 96.00 | 91.39 | 87.58 | 84.09 | 81.46 |
| TNA#2-T | 72.12 | 95.71 | 91.52 | 86.52 | 80.07 | 74.98 |
| TNA#2-N | 250.58 | 96.58 | 92.99 | 89.63 | 86.15 | 83.78 |
| TNA#3-T | 167.75 | 97.32 | 95.84 | 94.30 | 92.07 | 90.25 |
| TNA#3-N | 315.61 | 96.78 | 93.80 | 91.02 | 88.18 | 85.96 |
| TNA#4-T | 95.23 | 96.26 | 94.30 | 91.60 | 87.60 | 84.03 |
| TNA#4-N | 256.71 | 96.32 | 92.49 | 88.93 | 85.56 | 83.21 |
| TNA#5-T | 67.64 | 94.95 | 90.63 | 85.46 | 78.49 | 73.33 |
| TNA#5-N | 350.07 | 93.20 | 86.60 | 81.86 | 77.93 | 75.23 |
| TNA#6-T | 42.81 | 93.51 | 86.02 | 77.98 | 68.06 | 60.73 |
| TNA#6-N | 206.33 | 93.97 | 88.61 | 84.86 | 81.45 | 78.95 |
| TNA#7-T | 135.28 | 96.63 | 94.65 | 92.37 | 89.16 | 86.40 |
| TNA#7-N | 92.46 | 81.52 | 65.46 | 56.68 | 49.62 | 45.18 |
| TNA#8-T | 285.39 | 97.68 | 96.89 | 96.23 | 95.10 | 94.22 |
| TNA#8-N | 62.80 | 94.18 | 86.89 | 80.28 | 73.12 | 67.95 |
| TNA#9-T | 18.92 | 86.51 | 81.43 | 75.84 | 72.65 | 69.80 |
| TNA#9-N | 68.57 | 96.13 | 91.78 | 86.37 | 78.80 | 72.96 |
| TNA#10-T | 71.70 | 95.47 | 91.48 | 86.85 | 80.88 | 76.10 |
| TNA#10-N | 324.47 | 96.46 | 92.80 | 89.22 | 85.64 | 83.24 |
| TNA#11-T | 250.22 | 97.84 | 96.96 | 96.24 | 95.17 | 94.34 |
| TNA#11-N | 105.56 | 90.36 | 80.46 | 73.33 | 66.95 | 62.72 |
| TNA#12-T | 260.35 | 97.70 | 96.99 | 96.22 | 95.27 | 94.45 |
| TNA#12-N | 24.55 | 81.06 | 61.44 | 48.53 | 36.86 | 30.31 |
| TNA#13-T | 67.14 | 97.58 | 94.28 | 90.55 | 85.49 | 80.87 |
| TNA#13-N | 211.92 | 97.71 | 96.31 | 94.94 | 92.98 | 91.40 |
| TNA#14-T | 63.78 | 95.28 | 91.08 | 86.08 | 79.01 | 73.63 |
| TNA#14-N | 80.43 | 84.85 | 69.39 | 60.72 | 52.74 | 47.75 |
| TNA#15-T | 101.99 | 95.99 | 93.22 | 89.72 | 84.83 | 81.25 |
| TNA#15-N | 69.06 | 93.05 | 84.88 | 77.26 | 69.49 | 64.20 |
| TNA#16-T | 126.51 | 96.44 | 94.66 | 92.32 | 88.80 | 86.09 |
| TNA#16-N | 92.19 | 87.90 | 76.34 | 68.88 | 62.40 | 58.32 |

**Table S3.** Validation results using target capture sequencing and Sanger sequencing

| **Gene** | **Position** | **Exome  sequencing** | **Target capture  sequencing** | **Sanger sequencing** | **Validation** |
| --- | --- | --- | --- | --- | --- |
| *ADAMTS16* | chr5:5187877 | Positive | Positive | Positive | **O** |
| *AKAP10* | chr17:19823350 | Positive | Positive | Positive | **O** |
| *AKAP9* | chr7:91724351 | Positive | Positive | Positive | **O** |
| *AKT2* | chr19:40747961 | Positive | Positive | Positive | **O** |
| *AQP12B* | chr2:241622103 | Positive | - | Positive | **O** |
| *ARHGEF12* | chr11:120348991 | Positive | Positive | Positive | **O** |
| *ASPSCR1* | chr17:79954544 | Positive | - | Positive | **O** |
| *BRD2* | chr6:32945545 | Positive | Positive | Positive | **O** |
| *CASS4* | chr20:55027042 | Positive | Positive | Positive | **O** |
| *CCT2* | chr12:69981309 | Positive | Positive | Positive | **O** |
| *CD1A* | chr1:158225052 | Positive | Positive | Negative | **X** |
| *CDC27* | chr17:45234417 | Positive | Positive | Positive | **O** |
| *CDC27* | chr17:45234419 | Positive | Positive | Positive | **O** |
| *CHEK2* | chr22:29121233 | Positive | Positive | Positive | **O** |
| *COL27A1* | chr9:117071744 | Positive | - | Positive | **O** |
| *COL4A5* | chrX:107938522 | Positive | Positive | Positive | **O** |
| *CPSF2* | chr14:92621561 | Positive | Positive | Negative | **X** |
| *CSMD3* | chr8:113402923 | Positive | Positive | Positive | **O** |
| *CSPP1* | chr8:68005800 | Positive | Positive | Positive | **O** |
| *CTAGE4* | chr7:143882511 | Positive | - | Positive | **O** |
| *CTNNB1* | chr3:41266113 | Positive | Positive | Positive | **O** |
| *CUL3* | chr2:225365144 | Positive | Positive | Positive | **O** |
| *CYP2F1* | chr19:41633977 | Positive | Positive | Negative | **X** |
| *ERBB4* | chr2:212295795 | Positive | Positive | Positive | **O** |
| *ERC1* | chr12:1399135 | Positive | Positive | Positive | **O** |
| *GABRD* | chr1:1959067 | Positive | Positive | - | **O** |
| *GABRG1* | chr4:46053620 | Positive | Positive | - | **O** |
| *GABRG1* | chr4:46099344 | Positive | Positive | - | **O** |
| *GSTT2* | chr22:24325095 | Positive | - | Positive | **O** |
| *HELZ* | chr17:65105364 | Positive | Positive | Positive | **O** |
| *HIST1H4K* | chr6:27799008 | Positive | Positive | Negative | **X** |
| *HIVEP3* | chr1:42045651 | Positive | Positive | Positive | **O** |
| *HLA-B* | chr6:31324057 | Positive | Positive | Positive | **O** |
| *HOMER1* | chr5:78671893 | Positive | Positive | Positive | **O** |
| *HRNR* | chr1:152187437 | Positive | - | Positive | **O** |
| *H1FOO* | chr3:129262077 | Positive | - | Positive | **O** |
| *JPH1* | chr8:75171665 | Positive | Positive | Positive | **O** |
| *KIAA0895* | chr7:36373540 | Positive | Positive | Positive | **O** |
| *LDHAL6A* | chr11:18500358 | Positive | Positive | Positive | **O** |
| *MBD2* | chr18:51686266 | Positive | Positive | - | **O** |
| *MECP2* | chrX:153296461 | Positive | Positive | Positive | **O** |
| *MLL4* | chr19:36222857 | Positive | - | Positive | **O** |
| *MOSC1* | chr1:220970028 | Positive | - | Positive | **O** |
| *MST1R* | chr3:49924940 | Positive | Positive | Positive | **O** |
| *MTRF1L* | chr6:153323709 | Positive | Positive | Positive | **O** |
| *NAALAD2* | chr11:89880587 | Positive | Positive | Positive | **O** |
| *NBPF10* | chr1:145299932 | Positive | Positive | Positive | **O** |
| *NDRG1* | chr8:134260154 | Positive | Positive | Positive | **O** |
| *NOTCH2* | chr1:120611960 | Positive | - | Positive | **O** |
| *NUP50* | chr22:45574280 | Positive | Positive | Positive | **O** |
| *PABPC1* | chr8:101727750 | Positive | Positive | Positive | **O** |
| *PARP4* | chr13:25021323 | Positive | Positive | - | **O** |
| *PBRM1* | chr3:52643408 | Positive | Positive | Positive | **O** |
| *PCDHB14* | chr5:140605086 | Positive | Positive | - | **O (TNA#4)** |
| *PCDHB14* | chr5:140605086 | Positive | Positive | - | **O (TNA#6)** |
| *PCDHB15* | chr5:140627155 | Positive | Positive | - | **O** |
| *PHIP* | chr6:79735874 | Positive | Positive | Positive | **O** |
| *PIK3CA* | chr3:178917478 | Positive | Positive | Positive | **O** |
| *PLD5* | chr1:242271062 | Positive | Positive | Positive | **O** |
| *POTEB* | chr15:21071439 | Positive | - | Positive | **O** |
| *PRAMEF1* | chr1:12853509 | Positive | Positive | Positive | **O** |
| *PRRC1* | chr5:126860520 | Positive | Positive | Negative | **X** |
| *PRSS38* | chr1:228033197 | Positive | - | Positive | **O** |
| *PSPH* | chr7:56084929 | Positive | Positive | Positive | **O** |
| *PTPRC* | chr1:198685877 | Positive | Positive | Positive | **O** |
| *PTPRC* | chr1:198723415 | Positive | Positive | - | **O** |
| *RAD50* | chr5:131923749 | Positive | Positive | Positive | **O** |
| *RECQL5* | chr17:73647273 | Positive | Positive | Positive | **O** |
| *RFPL4A* | chr19:56273285 | Positive | Positive | Positive | **O** |
| *RIN3* | chr14:93118038 | Positive | - | Positive | **O** |
| *RNF213* | chr17:78317090 | Positive | Positive | Positive | **O** |
| *RPL19* | chr17:37360846 | Positive | Positive | Positive | **O** |
| *SEL1L2* | chr20:13936724 | Positive | Positive | Positive | **O** |
| *SLAIN2* | chr4:48381728 | Positive | Positive | Positive | **O** |
| *SLC7A1* | chr13:30107113 | Positive | Positive | Positive | **O** |
| *SOD2* | chr6:160103614 | Positive | Positive | Positive | **O** |
| *SULF1* | chr8:70512849 | Positive | Positive | Positive | **O** |
| *SYK* | chr9:93639869 | Positive | - | Positive | **O** |
| *SYNE2* | chr14:64554542 | Positive | Positive | Positive | **O** |
| *TCEB3* | chr1:24083477 | Positive | Positive | Positive | **O** |
| *TGFBR2* | chr3:30729914 | Positive | Positive | Positive | **O** |
| *TMTC1* | chr12:29709865 | Positive | Positive | Positive | **O** |
| *TP53BP1* | chr15:43748861 | Positive | Positive | Negative | **X** |
| *TP53BP1* | chr15:43738724 | Positive | Positive | Positive | **O** |
| *TPSD1* | chr16:1306523 | Positive | Positive | Positive | **O** |
| *TROAP* | chr12:49724304 | Positive | - | Positive | **O** |
| *TSC1* | chr9:135778003 | Positive | Positive | Positive | **O** |
| *UACA* | chr15:70991933 | Positive | Positive | Positive | **O** |
| *UNC93A* | chr6:167728775 | Positive | - | Positive | **O** |
| *USP29* | chr19:57641481 | Positive | Positive | Positive | **O** |
| *VCX2* | chrX:8138284 | Positive | Positive | Positive | **O** |
| *WAPAL* | chr10:88259559 | Positive | Positive | Positive | **O** |
| *WASH1* | chr9:16020 | Positive | Positive | Positive | **O** |
| *XBP1* | chr22:29191396 | Positive | Positive | Positive | **O** |
| *YTHDF1* | chr20:61833818 | Positive | Positive | Positive | **O (TNA#8)** |
| *YTHDF1* | chr20:61833818 | Positive | Positive | Positive | **O (TNA#10)** |
| *ZNF521* | chr18:22805549 | Positive | Positive | Positive | **O** |

**Table S4. Summary of validated somatic exonic mutations in *EGFR*/*KRAS*/*ALK*-negative lung adenocarcinomas**

|  |  |  |  |  |  |
| --- | --- | --- | --- | --- | --- |
| Patient | Mutation/Mb | Missense | Nonsense | Indel | Total |
| TNA#1 | 0.10 | 1 | 2 | 0 | 3 |
| TNA#2 | 0.10 | 3 | 0 | 0 | 3 |
| TNA#3 | 0.14 | 3 | 0 | 1 | 4 |
| TNA#4 | 0.17 | 4 | 1 | 0 | 5 |
| TNA#5 | 0.21 | 7 | 0 | 0 | 7 |
| TNA#6 | 0.21 | 7 | 0 | 0 | 7 |
| TNA#7 | 0.24 | 8 | 0 | 0 | 8 |
| TNA#8 | 0.31 | 7 | 1 | 1 | 9 |
| TNA#9 | 0.40 | 10 | 0 | 1 | 11 |
| TNA#10 | 0.40 | 10 | 0 | 1 | 11 |
| TNA#11 | 0.55 | 14 | 2 | 0 | 16 |
| TNA#12 | 0.58 | 18 | 0 | 0 | 18 |
| TNA#13 | 0.72 | 21 | 2 | 0 | 23 |
| TNA#14 | 0.87 | 21 | 4 | 0 | 25 |
| TNA#15 | 0.90 | 24 | 2 | 0 | 26 |
| TNA#16 | 0.94 | 26 | 2 | 0 | 28 |
|  |  |  |  |  |  |

**Table S5. Somatic mutations in *EGFR*/*KRAS*/*ALK*-negative lung adenocarcinoma exomes**

|  |  |  |  |  |  |  |  |  |  |  |  |  |  | missense | indel | nonsense | missense/ nonsense |
| --- | --- | --- | --- | --- | --- | --- | --- | --- | --- | --- | --- | --- | --- | --- | --- | --- | --- |
| **Gene** | **COSMIC v.65** | **TNA#1** | **TNA#2** | **TNA#3** | **TNA#4** | **TNA#5** | **TNA#6** | **TNA#7** | **TNA#8** | **TNA#9** | **TNA#10** | **TNA#11** | **TNA#12** | **TNA#13** | **TNA#14** | **TNA#15** | **TNA#16** |
| **Represented in Figure 1** |  |  |  |  |  |  |  |  |  |  |  |  |  |  |  |  |  |
| ***ERBB4*** | **O** |  |  |  |  |  |  |  |  |  |  |  | p.V840I |  |  |  |  |
| ***PIK3CA*** | **O** |  |  |  |  |  |  |  |  |  |  |  |  |  |  |  | p.G118D |
| ***AKT2*** | **-** |  |  |  |  |  |  |  |  |  |  |  |  | p.D153Y |  |  |  |
| ***TSC1*** | **-** |  |  |  |  |  |  |  |  |  |  |  |  |  | p.Q793* |  |  |
| ***CTNNB1*** | **O** |  | p.S37C |  |  |  |  |  |  |  |  |  |  |  |  |  |  |
| ***TGFBR2*** | **O** |  |  |  |  |  |  |  |  |  |  |  |  | p.R504W |  |  |  |
| ***SETD2*** | **-** |  |  |  |  |  |  |  |  |  | p.V1576F |  |  |  |  |  |  |
| ***PBRM1*** | **-** |  |  |  |  |  |  |  |  |  |  |  |  |  | p.E830* |  |  |
| ***MBD2*** | **-** |  |  |  |  |  |  |  |  |  |  |  |  |  |  |  | p.E373K |
| ***MECP2*** | **O** |  |  |  |  |  |  |  |  |  |  |  |  | p.G273V |  |  |  |
| ***CHEK2*** | **-** |  |  |  |  |  |  |  |  |  |  |  |  |  | p.R191G |  |  |
| ***CDC27*** | **O** |  |  |  |  |  |  | p.A273G |  |  |  |  | p.I235T |  |  |  |  |
| ***PARP4*** | **O** | 0 | 0 | 0 | 0 | 0 | 0 | 0 | 0 | 0 | 0 | 0 | p.I1039T | 0 | 0 | 0 | 0 |
| ***CUL3*** | **-** |  |  |  |  |  |  |  |  |  |  | p.Q516* |  |  |  |  |  |
| ***SOD2*** | **-** |  |  |  |  |  |  |  |  |  |  |  | p.K194E |  |  |  |  |
| ***GABRD*** | **-** |  |  |  |  |  |  |  |  | p.D174E |  |  |  |  |  |  |  |
| ***GABRG1*** | **-** |  |  |  |  |  |  |  |  | p.D43N |  |  |  | p.T318S |  |  |  |
| ***SYNE2*** | **-** | p.E3880* |  |  |  |  |  |  |  |  |  |  |  |  |  |  |  |
| ***NDRG1*** | **-** |  |  |  |  |  |  |  |  | p.L257fs |  |  |  |  |  |  |  |
| ***TFG*** | **-** |  |  |  | p.E240* |  |  |  |  |  |  |  |  |  |  |  |  |
| ***CSMD3*** | **-** |  |  |  |  |  |  |  |  |  |  |  |  | p.K1928N |  |  |  |
| ***SYK*** | **-** | 0 | 0 | p.A400S | 0 | 0 | 0 | 0 | 0 | 0 | 0 | 0 | 0 | 0 | 0 | 0 | 0 |
| ***PTPRC*** | **-** |  |  |  |  |  |  |  |  |  |  |  |  |  | p.T1176M |  | p.T453M |
| ***ASPSCR1*** | **-** |  |  |  |  |  |  |  |  |  |  |  |  |  | p.L252Q |  |  |
| ***YTHDF1*** | **-** |  |  |  |  |  |  |  | p.I492V |  | p.I492V |  |  |  |  |  |  |
| ***PCDHB14*** | **-** | 0 | 0 | 0 | p.Y670S | 0 | p.Y670S | 0 | 0 | 0 | 0 | 0 | 0 | 0 | 0 | 0 | 0 |
| ***PCDHB15*** | **-** | 0 | 0 | 0 | 0 | 0 | p.Y670S | 0 | 0 | 0 | 0 | 0 | 0 | 0 | 0 | 0 | 0 |
| **Others** |  |  |  |  |  |  |  |  |  |  |  |  |  |  |  |  |  |
| ***BDP1*** |  | nonsense | 0 | 0 | 0 | 0 | 0 | 0 | 0 | 0 | 0 | 0 | 0 | 0 | 0 | 0 | 0 |
| ***GSTT2*** |  | missense | 0 | 0 | 0 | 0 | 0 | 0 | 0 | 0 | 0 | 0 | 0 | 0 | 0 | 0 | 0 |
| ***PAPPA*** |  | 0 | missense | 0 | 0 | 0 | 0 | 0 | 0 | 0 | 0 | 0 | 0 | 0 | 0 | 0 | 0 |
| ***ZNF107*** |  | 0 | missense | 0 | 0 | 0 | 0 | 0 | 0 | 0 | 0 | 0 | 0 | 0 | 0 | 0 | 0 |
| ***MST1R*** |  | 0 | 0 | missense | 0 | 0 | 0 | 0 | 0 | 0 | 0 | 0 | 0 | 0 | 0 | 0 | 0 |
| ***HLA-B*** |  | 0 | 0 | missense | 0 | 0 | 0 | 0 | 0 | 0 | 0 | 0 | 0 | 0 | 0 | missense | 0 |
| ***NR1H2*** |  | 0 | 0 | indel | 0 | 0 | 0 | 0 | 0 | 0 | 0 | 0 | 0 | 0 | 0 | 0 | 0 |
| ***AQP12B*** |  | 0 | 0 | 0 | missense | 0 | missense | 0 | 0 | 0 | 0 | 0 | 0 | 0 | 0 | 0 | 0 |
| ***NBPF14*** |  | 0 | 0 | 0 | missense | 0 | 0 | 0 | 0 | 0 | 0 | 0 | 0 | 0 | 0 | 0 | 0 |
| ***VCX2*** |  | 0 | 0 | 0 | missense | 0 | 0 | 0 | 0 | 0 | 0 | 0 | 0 | 0 | 0 | 0 | 0 |
| ***CTAGE4*** |  | 0 | 0 | 0 | 0 | missense | 0 | 0 | missense | 0 | 0 | 0 | 0 | 0 | 0 | 0 | 0 |
| ***NBPF10*** |  | 0 | 0 | 0 | 0 | missense | 0 | 0 | 0 | 0 | 0 | 0 | 0 | 0 | 0 | 0 | 0 |
| ***LILRB4*** |  | 0 | 0 | 0 | 0 | missense | 0 | 0 | 0 | 0 | 0 | 0 | 0 | 0 | 0 | 0 | 0 |
| ***PLD5*** |  | 0 | 0 | 0 | 0 | missense | 0 | 0 | 0 | 0 | 0 | 0 | 0 | 0 | 0 | 0 | 0 |
| ***MCTP1*** |  | 0 | 0 | 0 | 0 | missense | 0 | 0 | 0 | 0 | 0 | 0 | 0 | 0 | 0 | 0 | 0 |
| ***WASH1*** |  | 0 | 0 | 0 | 0 | missense | 0 | 0 | 0 | 0 | 0 | 0 | 0 | 0 | 0 | missense | 0 |
| ***EXOC2*** |  | 0 | 0 | 0 | 0 | 0 | missense | 0 | 0 | 0 | 0 | 0 | 0 | 0 | 0 | 0 | 0 |
| ***RIN3*** |  | 0 | 0 | 0 | 0 | 0 | missense | 0 | 0 | 0 | 0 | 0 | 0 | 0 | 0 | 0 | 0 |
| ***TUBB2C*** |  | 0 | 0 | 0 | 0 | 0 | missense | 0 | 0 | 0 | 0 | 0 | 0 | 0 | 0 | 0 | 0 |
| ***TPSD1*** |  | 0 | 0 | 0 | 0 | 0 | missense | 0 | 0 | 0 | 0 | 0 | 0 | 0 | 0 | 0 | 0 |
| ***NOTCH2*** |  | 0 | 0 | 0 | 0 | 0 | 0 | missense | 0 | 0 | 0 | 0 | 0 | 0 | 0 | 0 | 0 |
| ***ZNF285*** |  | 0 | 0 | 0 | 0 | 0 | 0 | missense | 0 | 0 | 0 | 0 | 0 | 0 | 0 | 0 | 0 |
| ***TUBA3D*** |  | 0 | 0 | 0 | 0 | 0 | 0 | missense | 0 | 0 | 0 | 0 | 0 | 0 | 0 | 0 | 0 |
| ***TROAP*** |  | 0 | 0 | 0 | 0 | 0 | 0 | missense | 0 | 0 | 0 | 0 | 0 | 0 | 0 | 0 | 0 |
| ***H1FOO*** |  | 0 | 0 | 0 | 0 | 0 | 0 | missense | 0 | 0 | 0 | 0 | 0 | 0 | 0 | 0 | 0 |
| ***RFPL4A*** |  | 0 | 0 | 0 | 0 | 0 | 0 | missense | 0 | 0 | 0 | 0 | 0 | 0 | 0 | 0 | 0 |
| ***SNRNP200*** |  | 0 | 0 | 0 | 0 | 0 | 0 | 0 | nonsense | 0 | 0 | 0 | 0 | 0 | 0 | 0 | 0 |
| ***TPSAB1*** |  | 0 | 0 | 0 | 0 | 0 | 0 | 0 | missense | 0 | 0 | 0 | 0 | 0 | 0 | 0 | 0 |
| ***MICB*** |  | 0 | 0 | 0 | 0 | 0 | 0 | 0 | missense | 0 | 0 | 0 | 0 | 0 | 0 | 0 | 0 |
| ***MUC5AC*** |  | 0 | 0 | 0 | 0 | 0 | 0 | 0 | missense | 0 | 0 | 0 | 0 | 0 | 0 | 0 | 0 |
| ***C6orf134*** |  | 0 | 0 | 0 | 0 | 0 | 0 | 0 | missense | 0 | 0 | 0 | 0 | 0 | 0 | 0 | 0 |
| ***KEL*** |  | 0 | 0 | 0 | 0 | 0 | 0 | 0 | 0 | missense | 0 | 0 | 0 | 0 | 0 | 0 | 0 |
| ***ROBO4*** |  | 0 | 0 | 0 | 0 | 0 | 0 | 0 | 0 | missense | 0 | 0 | 0 | 0 | 0 | 0 | 0 |
| ***NBPF1*** |  | 0 | 0 | 0 | 0 | 0 | 0 | 0 | 0 | missense | 0 | 0 | 0 | 0 | 0 | 0 | 0 |
| ***SLC6A19*** |  | 0 | 0 | 0 | 0 | 0 | 0 | 0 | 0 | missense | 0 | 0 | 0 | 0 | 0 | 0 | 0 |
| ***ATP8B1*** |  | 0 | 0 | 0 | 0 | 0 | 0 | 0 | 0 | missense | 0 | 0 | 0 | 0 | 0 | 0 | 0 |
| ***UNC93A*** |  | 0 | 0 | 0 | 0 | 0 | 0 | 0 | 0 | missense | 0 | 0 | 0 | 0 | 0 | 0 | 0 |
| ***DMTF1*** |  | 0 | 0 | 0 | 0 | 0 | 0 | 0 | 0 | missense | 0 | 0 | 0 | 0 | 0 | 0 | 0 |
| ***PDIA2*** |  | 0 | 0 | 0 | 0 | 0 | 0 | 0 | 0 | missense | 0 | 0 | 0 | 0 | 0 | 0 | 0 |
| ***JPH1*** |  | 0 | 0 | 0 | 0 | 0 | 0 | 0 | 0 | 0 | missense | 0 | 0 | 0 | 0 | 0 | 0 |
| ***PRAMEF1*** |  | 0 | 0 | 0 | 0 | 0 | 0 | 0 | 0 | 0 | missense | 0 | 0 | 0 | 0 | 0 | 0 |
| ***OR1L4*** |  | 0 | 0 | 0 | 0 | 0 | 0 | 0 | 0 | 0 | missense | 0 | 0 | 0 | 0 | 0 | 0 |
| ***HOXD9*** |  | 0 | 0 | 0 | 0 | 0 | 0 | 0 | 0 | 0 | indel | 0 | 0 | 0 | 0 | 0 | 0 |
| ***TEKT4*** |  | 0 | 0 | 0 | 0 | 0 | 0 | 0 | 0 | 0 | missense | 0 | 0 | 0 | 0 | 0 | 0 |
| ***MTRF1L*** |  | 0 | 0 | 0 | 0 | 0 | 0 | 0 | 0 | 0 | missense | 0 | 0 | missense | 0 | 0 | 0 |
| ***MLL4*** |  | 0 | 0 | 0 | 0 | 0 | 0 | 0 | 0 | 0 | missense | 0 | 0 | 0 | 0 | 0 | 0 |
| ***TBC1D26*** |  | 0 | 0 | 0 | 0 | 0 | 0 | 0 | 0 | 0 | missense | 0 | 0 | 0 | 0 | 0 | 0 |
| ***POTEB*** |  | 0 | 0 | 0 | 0 | 0 | 0 | 0 | 0 | 0 | missense | 0 | 0 | 0 | 0 | 0 | 0 |
| ***ZNF521*** |  | 0 | 0 | 0 | 0 | 0 | 0 | 0 | 0 | 0 | 0 | missense | 0 | 0 | 0 | 0 | 0 |
| ***USP29*** |  | 0 | 0 | 0 | 0 | 0 | 0 | 0 | 0 | 0 | 0 | missense | 0 | 0 | 0 | 0 | 0 |
| ***GOLGB1*** |  | 0 | 0 | 0 | 0 | 0 | 0 | 0 | 0 | 0 | 0 | missense | 0 | 0 | 0 | 0 | 0 |
| ***SLC7A1*** |  | 0 | 0 | 0 | 0 | 0 | 0 | 0 | 0 | 0 | 0 | missense | 0 | 0 | 0 | 0 | 0 |
| ***UACA*** |  | 0 | 0 | 0 | 0 | 0 | 0 | 0 | 0 | 0 | 0 | missense | 0 | 0 | 0 | 0 | 0 |
| ***SULF1*** |  | 0 | 0 | 0 | 0 | 0 | 0 | 0 | 0 | 0 | 0 | missense | 0 | 0 | 0 | 0 | 0 |
| ***OR51E2*** |  | 0 | 0 | 0 | 0 | 0 | 0 | 0 | 0 | 0 | 0 | missense | 0 | 0 | 0 | 0 | 0 |
| ***COL27A1*** |  | 0 | 0 | 0 | 0 | 0 | 0 | 0 | 0 | 0 | 0 | missense | 0 | 0 | 0 | 0 | 0 |
| ***PABPC1*** |  | 0 | 0 | 0 | 0 | 0 | 0 | 0 | 0 | 0 | 0 | missense | 0 | 0 | 0 | 0 | 0 |
| ***OR10C1*** |  | 0 | 0 | 0 | 0 | 0 | 0 | 0 | 0 | 0 | 0 | missense | 0 | 0 | 0 | 0 | 0 |
| ***C4orf41*** |  | 0 | 0 | 0 | 0 | 0 | 0 | 0 | 0 | 0 | 0 | missense | 0 | 0 | 0 | 0 | 0 |
| ***AP3M1*** |  | 0 | 0 | 0 | 0 | 0 | 0 | 0 | 0 | 0 | 0 | missense | 0 | 0 | 0 | 0 | 0 |
| ***HLA-DQB2*** |  | 0 | 0 | 0 | 0 | 0 | 0 | 0 | 0 | 0 | 0 | missense | 0 | 0 | 0 | 0 | 0 |
| ***CCT2*** |  | 0 | 0 | 0 | 0 | 0 | 0 | 0 | 0 | 0 | 0 | nonsense | 0 | 0 | 0 | 0 | 0 |
| ***NFKBID*** |  | 0 | 0 | 0 | 0 | 0 | 0 | 0 | 0 | 0 | 0 | missense | 0 | 0 | 0 | 0 | 0 |
| ***C11orf41*** |  | 0 | 0 | 0 | 0 | 0 | 0 | 0 | 0 | 0 | 0 | 0 | missense | 0 | 0 | 0 | 0 |
| ***HUWE1*** |  | 0 | 0 | 0 | 0 | 0 | 0 | 0 | 0 | 0 | 0 | 0 | missense | 0 | 0 | 0 | 0 |
| ***PLXNA2*** |  | 0 | 0 | 0 | 0 | 0 | 0 | 0 | 0 | 0 | 0 | 0 | missense | 0 | 0 | 0 | 0 |
| ***TANC2*** |  | 0 | 0 | 0 | 0 | 0 | 0 | 0 | 0 | 0 | 0 | 0 | missense | 0 | 0 | 0 | 0 |
| ***PHIP*** |  | 0 | 0 | 0 | 0 | 0 | 0 | 0 | 0 | 0 | 0 | 0 | missense | 0 | 0 | 0 | 0 |
| ***NAALAD2*** |  | 0 | 0 | 0 | 0 | 0 | 0 | 0 | 0 | 0 | 0 | 0 | missense | 0 | 0 | 0 | 0 |
| ***MOV10L1*** |  | 0 | 0 | 0 | 0 | 0 | 0 | 0 | 0 | 0 | 0 | 0 | missense | 0 | 0 | 0 | 0 |
| ***EXOSC9*** |  | 0 | 0 | 0 | 0 | 0 | 0 | 0 | 0 | 0 | 0 | 0 | missense | 0 | 0 | 0 | 0 |
| ***HLA-DRB1*** |  | 0 | 0 | 0 | 0 | 0 | 0 | 0 | 0 | 0 | 0 | 0 | missense | 0 | 0 | 0 | 0 |
| ***PLA2G3*** |  | 0 | 0 | 0 | 0 | 0 | 0 | 0 | 0 | 0 | 0 | 0 | missense | 0 | 0 | 0 | 0 |
| ***RNASEH2A*** |  | 0 | 0 | 0 | 0 | 0 | 0 | 0 | 0 | 0 | 0 | 0 | missense | 0 | 0 | 0 | 0 |
| ***MUC16*** |  | 0 | 0 | 0 | 0 | 0 | 0 | 0 | 0 | 0 | 0 | 0 | missense | missense | 0 | 0 | 0 |
| ***COL4A5*** |  | 0 | 0 | 0 | 0 | 0 | 0 | 0 | 0 | 0 | 0 | 0 | 0 | missense | 0 | 0 | 0 |
| ***TMTC1*** |  | 0 | 0 | 0 | 0 | 0 | 0 | 0 | 0 | 0 | 0 | 0 | 0 | missense | 0 | 0 | 0 |
| ***LILRB2*** |  | 0 | 0 | 0 | 0 | 0 | 0 | 0 | 0 | 0 | 0 | 0 | 0 | missense | 0 | 0 | 0 |
| ***ADAMTS7*** |  | 0 | 0 | 0 | 0 | 0 | 0 | 0 | 0 | 0 | 0 | 0 | 0 | missense | 0 | 0 | 0 |
| ***GFRA1*** |  | 0 | 0 | 0 | 0 | 0 | 0 | 0 | 0 | 0 | 0 | 0 | 0 | missense | 0 | 0 | 0 |
| ***CSPP1*** |  | 0 | 0 | 0 | 0 | 0 | 0 | 0 | 0 | 0 | 0 | 0 | 0 | missense | 0 | 0 | 0 |
| ***OSGEP*** |  | 0 | 0 | 0 | 0 | 0 | 0 | 0 | 0 | 0 | 0 | 0 | 0 | missense | 0 | 0 | 0 |
| ***CASS4*** |  | 0 | 0 | 0 | 0 | 0 | 0 | 0 | 0 | 0 | 0 | 0 | 0 | missense | 0 | 0 | 0 |
| ***DNPEP*** |  | 0 | 0 | 0 | 0 | 0 | 0 | 0 | 0 | 0 | 0 | 0 | 0 | missense | 0 | 0 | 0 |
| ***TMEM86A*** |  | 0 | 0 | 0 | 0 | 0 | 0 | 0 | 0 | 0 | 0 | 0 | 0 | missense | 0 | 0 | 0 |
| ***CXorf58*** |  | 0 | 0 | 0 | 0 | 0 | 0 | 0 | 0 | 0 | 0 | 0 | 0 | missense | 0 | 0 | 0 |
| ***CRYBA4*** |  | 0 | 0 | 0 | 0 | 0 | 0 | 0 | 0 | 0 | 0 | 0 | 0 | nonsense | 0 | 0 | 0 |
| ***ANKRD31*** |  | 0 | 0 | 0 | 0 | 0 | 0 | 0 | 0 | 0 | 0 | 0 | 0 | missense | 0 | 0 | 0 |
| ***APOB*** |  | 0 | 0 | 0 | 0 | 0 | 0 | 0 | 0 | 0 | 0 | 0 | 0 | 0 | missense | 0 | 0 |
| ***FER1L6*** |  | 0 | 0 | 0 | 0 | 0 | 0 | 0 | 0 | 0 | 0 | 0 | 0 | 0 | missense | 0 | 0 |
| ***AKAP9*** |  | 0 | 0 | 0 | 0 | 0 | 0 | 0 | 0 | 0 | 0 | 0 | 0 | 0 | missense | 0 | 0 |
| ***OR6K6*** |  | 0 | 0 | 0 | 0 | 0 | 0 | 0 | 0 | 0 | 0 | 0 | 0 | 0 | missense | 0 | 0 |
| ***C7orf10*** |  | 0 | 0 | 0 | 0 | 0 | 0 | 0 | 0 | 0 | 0 | 0 | 0 | 0 | missense | 0 | 0 |
| ***TP53BP1*** |  | 0 | 0 | 0 | 0 | 0 | 0 | 0 | 0 | 0 | 0 | 0 | 0 | 0 | missense | 0 | 0 |
| ***ZSWIM5*** |  | 0 | 0 | 0 | 0 | 0 | 0 | 0 | 0 | 0 | 0 | 0 | 0 | 0 | missense | 0 | 0 |
| ***AKAP10*** |  | 0 | 0 | 0 | 0 | 0 | 0 | 0 | 0 | 0 | 0 | 0 | 0 | 0 | missense | 0 | 0 |
| ***SEL1L2*** |  | 0 | 0 | 0 | 0 | 0 | 0 | 0 | 0 | 0 | 0 | 0 | 0 | 0 | missense | 0 | 0 |
| ***WAPAL*** |  | 0 | 0 | 0 | 0 | 0 | 0 | 0 | 0 | 0 | 0 | 0 | 0 | 0 | missense | 0 | 0 |
| ***ZC3H18*** |  | 0 | 0 | 0 | 0 | 0 | 0 | 0 | 0 | 0 | 0 | 0 | 0 | 0 | missense | 0 | 0 |
| ***HOMER1*** |  | 0 | 0 | 0 | 0 | 0 | 0 | 0 | 0 | 0 | 0 | 0 | 0 | 0 | missense | 0 | 0 |
| ***KIAA0895*** |  | 0 | 0 | 0 | 0 | 0 | 0 | 0 | 0 | 0 | 0 | 0 | 0 | 0 | missense | 0 | 0 |
| ***SGCE*** |  | 0 | 0 | 0 | 0 | 0 | 0 | 0 | 0 | 0 | 0 | 0 | 0 | 0 | missense | 0 | 0 |
| ***CDC23*** |  | 0 | 0 | 0 | 0 | 0 | 0 | 0 | 0 | 0 | 0 | 0 | 0 | 0 | missense | 0 | 0 |
| ***SLAIN2*** |  | 0 | 0 | 0 | 0 | 0 | 0 | 0 | 0 | 0 | 0 | 0 | 0 | 0 | missense | 0 | 0 |
| ***RAB41*** |  | 0 | 0 | 0 | 0 | 0 | 0 | 0 | 0 | 0 | 0 | 0 | 0 | 0 | missense | 0 | 0 |
| ***TCEB3*** |  | 0 | 0 | 0 | 0 | 0 | 0 | 0 | 0 | 0 | 0 | 0 | 0 | 0 | missense | 0 | 0 |
| ***SHB*** |  | 0 | 0 | 0 | 0 | 0 | 0 | 0 | 0 | 0 | 0 | 0 | 0 | 0 | nonsense | 0 | 0 |
| ***HRNR*** |  | 0 | 0 | 0 | 0 | 0 | 0 | 0 | 0 | 0 | 0 | 0 | 0 | 0 | 0 | missense | 0 |
| ***ADCY8*** |  | 0 | 0 | 0 | 0 | 0 | 0 | 0 | 0 | 0 | 0 | 0 | 0 | 0 | 0 | missense | 0 |
| ***GRIA2*** |  | 0 | 0 | 0 | 0 | 0 | 0 | 0 | 0 | 0 | 0 | 0 | 0 | 0 | 0 | missense | 0 |
| ***RNF213*** |  | 0 | 0 | 0 | 0 | 0 | 0 | 0 | 0 | 0 | 0 | 0 | 0 | 0 | 0 | missense | 0 |
| ***MYO10*** |  | 0 | 0 | 0 | 0 | 0 | 0 | 0 | 0 | 0 | 0 | 0 | 0 | 0 | 0 | missense | 0 |
| ***HELZ*** |  | 0 | 0 | 0 | 0 | 0 | 0 | 0 | 0 | 0 | 0 | 0 | 0 | 0 | 0 | nonsense | 0 |
| ***PRSS38*** |  | 0 | 0 | 0 | 0 | 0 | 0 | 0 | 0 | 0 | 0 | 0 | 0 | 0 | 0 | missense | 0 |
| ***KARS*** |  | 0 | 0 | 0 | 0 | 0 | 0 | 0 | 0 | 0 | 0 | 0 | 0 | 0 | 0 | missense | 0 |
| ***GPA33*** |  | 0 | 0 | 0 | 0 | 0 | 0 | 0 | 0 | 0 | 0 | 0 | 0 | 0 | 0 | missense | 0 |
| ***RBM46*** |  | 0 | 0 | 0 | 0 | 0 | 0 | 0 | 0 | 0 | 0 | 0 | 0 | 0 | 0 | missense | 0 |
| ***ERC1*** |  | 0 | 0 | 0 | 0 | 0 | 0 | 0 | 0 | 0 | 0 | 0 | 0 | 0 | 0 | missense | 0 |
| ***KLHL22*** |  | 0 | 0 | 0 | 0 | 0 | 0 | 0 | 0 | 0 | 0 | 0 | 0 | 0 | 0 | missense | 0 |
| ***MOSC1*** |  | 0 | 0 | 0 | 0 | 0 | 0 | 0 | 0 | 0 | 0 | 0 | 0 | 0 | 0 | missense | 0 |
| ***RPL19*** |  | 0 | 0 | 0 | 0 | 0 | 0 | 0 | 0 | 0 | 0 | 0 | 0 | 0 | 0 | missense | 0 |
| ***FANK1*** |  | 0 | 0 | 0 | 0 | 0 | 0 | 0 | 0 | 0 | 0 | 0 | 0 | 0 | 0 | nonsense | 0 |
| ***CC2D2B*** |  | 0 | 0 | 0 | 0 | 0 | 0 | 0 | 0 | 0 | 0 | 0 | 0 | 0 | 0 | missense | 0 |
| ***SLC2A13*** |  | 0 | 0 | 0 | 0 | 0 | 0 | 0 | 0 | 0 | 0 | 0 | 0 | 0 | 0 | missense | 0 |
| ***ZNF488*** |  | 0 | 0 | 0 | 0 | 0 | 0 | 0 | 0 | 0 | 0 | 0 | 0 | 0 | 0 | missense | 0 |
| ***FRG1*** |  | 0 | 0 | 0 | 0 | 0 | 0 | 0 | 0 | 0 | 0 | 0 | 0 | 0 | 0 | missense | 0 |
| ***ARHGEF12*** |  | 0 | 0 | 0 | 0 | 0 | 0 | 0 | 0 | 0 | 0 | 0 | 0 | 0 | 0 | missense | 0 |
| ***SRP14*** |  | 0 | 0 | 0 | 0 | 0 | 0 | 0 | 0 | 0 | 0 | 0 | 0 | 0 | 0 | missense | 0 |
| ***RECQL5*** |  | 0 | 0 | 0 | 0 | 0 | 0 | 0 | 0 | 0 | 0 | 0 | 0 | 0 | 0 | missense | 0 |
| ***FOXK2*** |  | 0 | 0 | 0 | 0 | 0 | 0 | 0 | 0 | 0 | 0 | 0 | 0 | 0 | 0 | missense | 0 |
| ***NUP50*** |  | 0 | 0 | 0 | 0 | 0 | 0 | 0 | 0 | 0 | 0 | 0 | 0 | 0 | 0 | missense | 0 |
| ***USH2A*** |  | 0 | 0 | 0 | 0 | 0 | 0 | 0 | 0 | 0 | 0 | 0 | 0 | 0 | 0 | 0 | missense |
| ***SVEP1*** |  | 0 | 0 | 0 | 0 | 0 | 0 | 0 | 0 | 0 | 0 | 0 | 0 | 0 | 0 | 0 | missense |
| ***ADAMTS16*** |  | 0 | 0 | 0 | 0 | 0 | 0 | 0 | 0 | 0 | 0 | 0 | 0 | 0 | 0 | 0 | missense |
| ***HIVEP3*** |  | 0 | 0 | 0 | 0 | 0 | 0 | 0 | 0 | 0 | 0 | 0 | 0 | 0 | 0 | 0 | nonsense |
| ***AMOT*** |  | 0 | 0 | 0 | 0 | 0 | 0 | 0 | 0 | 0 | 0 | 0 | 0 | 0 | 0 | 0 | missense |
| ***OR2G2*** |  | 0 | 0 | 0 | 0 | 0 | 0 | 0 | 0 | 0 | 0 | 0 | 0 | 0 | 0 | 0 | missense |
| ***PTPN5*** |  | 0 | 0 | 0 | 0 | 0 | 0 | 0 | 0 | 0 | 0 | 0 | 0 | 0 | 0 | 0 | nonsense |
| ***RAD50*** |  | 0 | 0 | 0 | 0 | 0 | 0 | 0 | 0 | 0 | 0 | 0 | 0 | 0 | 0 | 0 | missense |
| ***PDE5A*** |  | 0 | 0 | 0 | 0 | 0 | 0 | 0 | 0 | 0 | 0 | 0 | 0 | 0 | 0 | 0 | missense |
| ***TRDMT1*** |  | 0 | 0 | 0 | 0 | 0 | 0 | 0 | 0 | 0 | 0 | 0 | 0 | 0 | 0 | 0 | missense |
| ***CNGA1*** |  | 0 | 0 | 0 | 0 | 0 | 0 | 0 | 0 | 0 | 0 | 0 | 0 | 0 | 0 | 0 | missense |
| ***C12orf41*** |  | 0 | 0 | 0 | 0 | 0 | 0 | 0 | 0 | 0 | 0 | 0 | 0 | 0 | 0 | 0 | missense |
| ***C14orf39*** |  | 0 | 0 | 0 | 0 | 0 | 0 | 0 | 0 | 0 | 0 | 0 | 0 | 0 | 0 | 0 | missense |
| ***KIAA0355*** |  | 0 | 0 | 0 | 0 | 0 | 0 | 0 | 0 | 0 | 0 | 0 | 0 | 0 | 0 | 0 | missense |
| ***XBP1*** |  | 0 | 0 | 0 | 0 | 0 | 0 | 0 | 0 | 0 | 0 | 0 | 0 | 0 | 0 | 0 | missense |
| ***USP39*** |  | 0 | 0 | 0 | 0 | 0 | 0 | 0 | 0 | 0 | 0 | 0 | 0 | 0 | 0 | 0 | missense |
| ***MAGEA10*** |  | 0 | 0 | 0 | 0 | 0 | 0 | 0 | 0 | 0 | 0 | 0 | 0 | 0 | 0 | 0 | missense |
| ***LCE1B*** |  | 0 | 0 | 0 | 0 | 0 | 0 | 0 | 0 | 0 | 0 | 0 | 0 | 0 | 0 | 0 | missense |
| ***BRD2*** |  | 0 | 0 | 0 | 0 | 0 | 0 | 0 | 0 | 0 | 0 | 0 | 0 | 0 | 0 | 0 | missense |
| ***LDHAL6A*** |  | 0 | 0 | 0 | 0 | 0 | 0 | 0 | 0 | 0 | 0 | 0 | 0 | 0 | 0 | 0 | missense |
| ***SMCR8*** |  | 0 | 0 | 0 | 0 | 0 | 0 | 0 | 0 | 0 | 0 | 0 | 0 | 0 | 0 | 0 | missense |
| ***PSPH*** |  | 0 | 0 | 0 | 0 | 0 | 0 | 0 | 0 | 0 | 0 | 0 | 0 | 0 | 0 | 0 | missense |
| ***ME1*** |  | 0 | 0 | 0 | 0 | 0 | 0 | 0 | 0 | 0 | 0 | 0 | 0 | 0 | 0 | 0 | missense |
| ***CCDC66*** |  | 0 | 0 | 0 | 0 | 0 | 0 | 0 | 0 | 0 | 0 | 0 | 0 | 0 | 0 | 0 | indel |
| ***MTCH2*** |  | 0 | 0 | 0 | 0 | 0 | 0 | 0 | 0 | 0 | 0 | 0 | 0 | 0 | 0 | 0 | missense |

**Table S6. Mutated genes and loci information in *EGFR*/*KRAS*-negative lung adenocarcinoma.**

| Gene | Mutation frequency in our set (N=16) | Mutation frequency in independent set (N=40) | Mutation frequency in TCGA set  (N=14) | Total (N) | AA substitution | COSMIC v.65 | No. of damage algorithms |
| --- | --- | --- | --- | --- | --- | --- | --- |
| *TP53* | - | 6/40 | 2/14 | 8/70 | K93R 122_122del Y124C C203F G206S R209W E219* G295A | O O O O O O O O | 4/4 NA 4/4 4/4 4/4 4/4 2/4 4/4 |
| *SETD2* | 1/16 | 2/40 | 1/14 | 4/70 | R839* V1576F Q1981* K2067* | - - - - | 1/2 3/3 2/2 0/1 |
| *CSMD3* | 1/16 | 1/40 | 2/14 | 4/70 | P667S M1440I K1928N Y2028C | O - - - | 3/4 3/4 3/4 3/4 |
| *PTPRC* | 2/16 | 1/40 | - | 3/70 | Y444N T453M T1176M | O - - | 3/4 3/4 4/4 |
| *SYNE2* | 1/16 | 2/40 | - | 3/70 | 2579_2580del E3880* E3903K | - - O | NA NA 3/4 |
| *GRIN2A* | - | 3/40 | - | 3/70 | L307Q N886S T1069M | - O O | 3/4 0/4 3/4 |
| *CDH10* | - | 2/40 | 1/14 | 3/70 | E171K D315H R472C | O O O | 4/4 1/4 4/4 |
| *ERBB2* | - | - | 3/14 | 3/70 | E740delinsEAYVM (2) G746delinsVC | - O | NA NA |
| *SMAD4* | - | - | 3/14 | 3/70 | S242* R361S D493N | - O O | 1/2 4/4 3/4 |
| *CDC27* | 2/16 | - | - | 2/70 | I235T A273G | - O | 4/4 3/4 |
| *GABRG1* | 2/16 | - | - | 2/70 | D43N T318S | - - | 2/4 3/4 |
| *YTHDF1* | 2/16 | - | - | 2/70 | I492V (2) | - | 4/4 |
| *PCDHB14* | 2/16 | - | - | 2/70 | Y670S (2) | - | 2/3 |
| *MECP2* | 1/16 | - | 1/14 | 2/70 | R168L G273V | - O | 2/3 3/3 |
| *NRAS* | - | 2/40 | - | 2/70 | Q61H Q61K | O O | 2/4 3/4 |
| *MET* | - | - | 2/14 | 2/70 | Y1021* 1027_1028del | - - | 2/2 NA |
| *EPHA2* | - | 2/40 | - | 2/70 | P147L P278S | O O | 4/4 2/4 |
| *CHD2* | - | 2/40 | - | 2/70 | S391fs splicing(c.5153) | - O | NA NA |
| *ERBB4* | 1/16 | - | - | 1/70 | V840I | O | 4/4 |
| *PIK3CA* | 1/16 | - | - | 1/70 | G118D | O | 2/3 |
| *AKT2* | 1/16 | - | - | 1/70 | D153Y | - | 4/4 |
| *TSC1* | 1/16 | - | - | 1/70 | Q793* | - | 2/2 |
| *CTNNB1* | 1/16 | - | - | 1/70 | S37C | O | 4/4 |
| *TGFBR2* | 1/16 | - | - | 1/70 | R504W | O | 4/4 |
| *PBRM1* | 1/16 | - | - | 1/70 | E830* | - | 2/2 |
| *MBD2* | 1/16 | - | - | 1/70 | E373K | - | 4/4 |
| *CHEK2* | 1/16 | - | - | 1/70 | R191G | - | 3/4 |
| *PARP4* | 1/16 | - | - | 1/70 | I1039T | O | 4/4 |
| *CUL3* | 1/16 | - | - | 1/70 | Q516* | - | 2/2 |
| *SOD2* | 1/16 | - | - | 1/70 | K194E | - | 3/4 |
| *GABRD* | 1/16 | - | - | 1/70 | D174E | - | 4/4 |
| *NDRG1* | 1/16 | - | - | 1/70 | L257fs | - | NA |
| *TFG* | 1/16 | - | - | 1/70 | E240* | - | 1/1 |
| *SYK* | 1/16 | - | - | 1/70 | A400S | - | 4/4 |
| *ASPSCR1* | 1/16 | - | - | 1/70 | L252Q | - | 3/3 |
| *PCDHB15* | 1/16 | - | - | 1/70 | Y670S | - | 3/3 |
| *NUP98* | - | 2/40 | 1/14 | 3/70 | G716C S1294C A1596V | - O - | 4/4 3/3 1/3 |
| *USP39* | 1/16 | 2/40 | - | 3/70 | D119Y Q563* 329_329del | - O - | 3/3 0/1 NA |
| *SLITRK2* | - | 2/40 | - | 2/70 | S276fs N554H | O O | NA 3/4 |
| *TAF1* | - | 2/40 | - | 2/70 | R1442W Y1194D | O O | 4/4 4/4 |
| *UNC13B* | - | 2/40 | - | 2/70 | T638I G500D | O O | 3/4 4/4 |
| *FN1* | - | 2/40 | - | 2/70 | D920G V2171fs | - O | 4/4 NA |
| *NAV2* | - | 2/40 | - | 2/70 | R496L S1413R | O O | 4/4 4/4 |
| *NUP133* | - | 2/40 | - | 2/70 | S189R 1050_1051del | O - | 4/4 NA |
| *CDC42BPG* | - | 2/40 | - | 2/70 | G358E P925T | O O | 3/4 3/4 |
| *COL25A1* | - | - | 2/14 | 2/70 | R375Q D289N | O O | 3/3 2/3 |
| *GRM1* | - | - | 2/14 | 2/70 | S228F S207C | - - | 4/4 4/4 |
| *ACACB* | - | - | 2/14 | 2/70 | S2314Y L2182fs | - - | 2/3 NA |
| *ANKHD1-EIF4EBP3* | - | - | 2/14 | 2/70 | D286N 1532_1532del | - - | 2/3 NA |
| *ARSD* | - | - | 2/14 | 2/70 | C583Y splicing(c.1136) | - - | 3/4 NA |
| *CRIPAK* | - | - | 2/14 | 2/70 | R430C 153_163del | O O | 2/3 NA |
| *GRID1* | - | - | 2/14 | 2/70 | R462H K694N | - - | 4/4 3/4 |
| *HMCN1* | - | - | 2/14 | 2/70 | A2271V G3137* | - - | 3/4 2/2 |
| *MST1* | - | - | 2/14 | 2/70 | 406_407del R535G | - - | NA 3/4 |
| *SPAM1* | - | - | 2/14 | 2/70 | R211W W339C | - - | 4/4 4/4 |
| *TDP2* | - | - | 2/14 | 2/70 | W284L L342P | - - | 4/4 4/4 |
| *GOLGB1* | 1/16 | 1/40 | - | 2/70 | E85D V2705L | - O | 3/4 3/4 |
| *DNPEP* | 1/16 | 1/40 | - | 2/70 | P422L G102W | - O | 3/4 4/4 |

**Table S7.** Sequences of molecular inversion probes (MIPs)

A preview of Table S7 is shown below.

The full version is available at *‘Supple_Tables.xls’. –‘Table_S7’*.


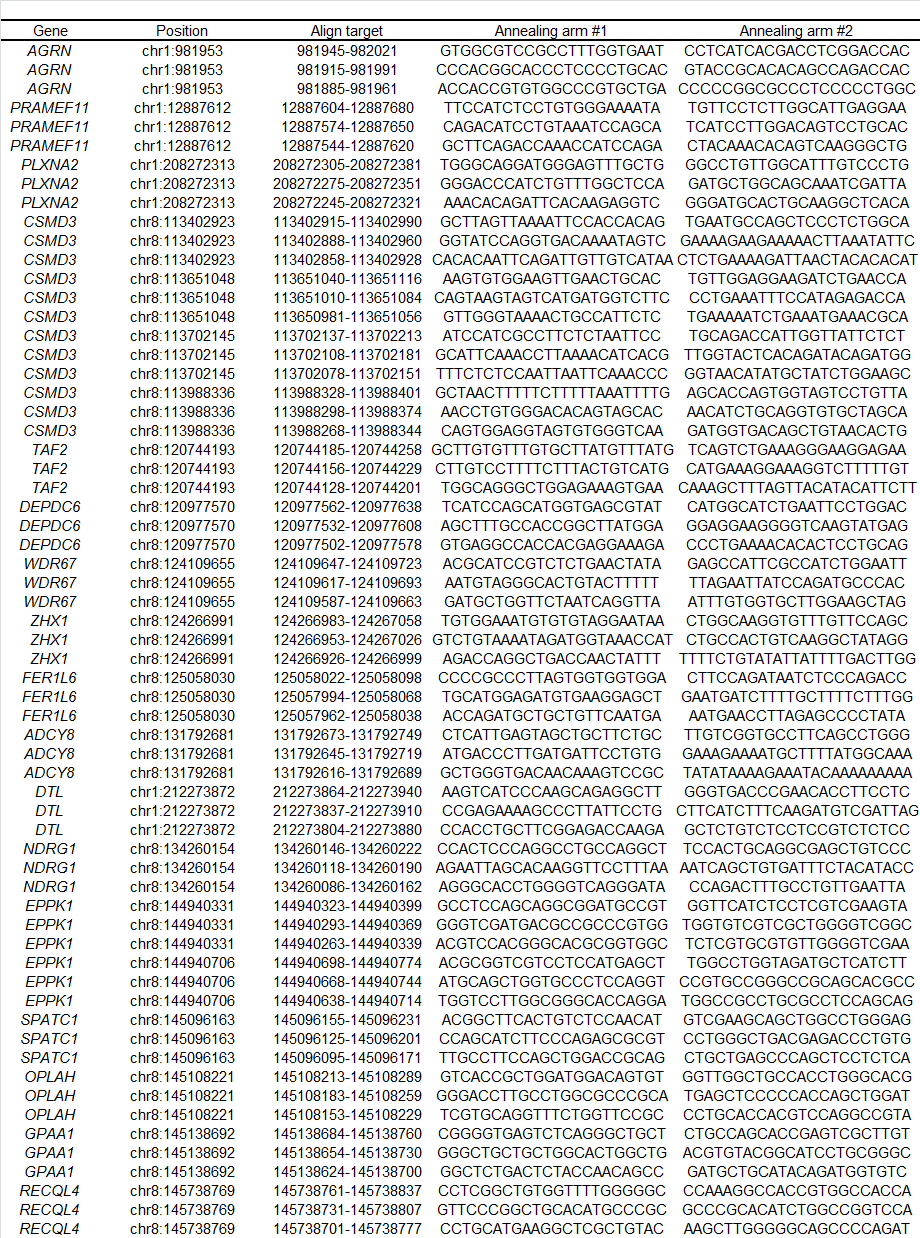


**Table S8.** Sequences of primers used for Sanger sequencing.

A preview of Table S8 is shown below.

The full version is available at *‘Supple_Tables.xls’. –‘Table_S8’*.


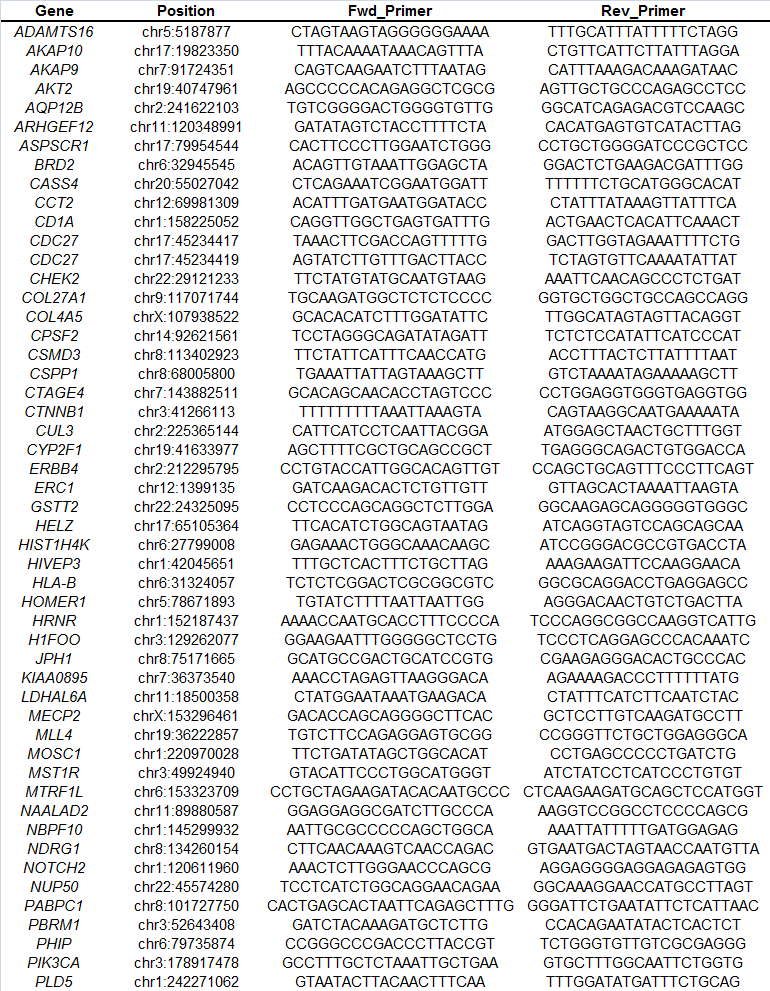


**References for exome analysis**

Novoalign ([http://novocraft.com](http://novocraft.com/)) – for alignment of sequencing data

SAMTools – for manipulating Sequence Alignment/Map (SAM) format

Li H, Handsaker B, Wysoker A, Fennell T, Ruan J, Homer N, Marth G, Abecasis G, Durbin R. 2009. The Sequence Alignment/Map format and SAMtools*. Bioinformatics* **25**(16): 2078-2079.

Picard ([http://picard.sourceforge.net](http://picard.sourceforge.net/)) – for manipulation of aligned sequencing data (FixMateInformation, MarkDuplication)

GATK (Genome Analysis Toolkit; version 1.4-21) – for detection of genetic variants in exome data

McKenna A, Hanna M, Banks E, Sivachenko A, Cibulskis K, Kernytsky A, Garimella K, Altshuler D, Gabriel S, Daly M et al. 2010. The Genome Analysis Toolkit: a MapReduce framework for analyzing next-generation DNA sequencing data. *Genome research* **20**(9): 1297-1303.

DePristo MA, Banks E, Poplin R, Garimella KV, Maguire JR, Hartl C, Philippakis AA, del Angel G, Rivas MA, Hanna M et al. 2011. A framework for variation discovery and genotyping using next-generation DNA sequencing data. *Nature genetics* **43**(5): 491-498.

muTect – for detection of genetic variants in exome data

Cibulskis K, Lawrence MS, Carter SL, Sivachenko A, Jaffe D, Sougnez C, Gabriel S, Meyerson M, Lander ES, Getz G. 2013. Sensitive detection of somatic point mutations in impure and heterogeneous cancer samples. *Nature biotechnology* **31**(3): 213-219.

VarScan – for detection of genetic variants in exome data

Koboldt DC, Chen K, Wylie T, Larson DE, McLellan MD, Mardis ER, Weinstock GM, Wilson RK, Ding L. 2009. VarScan: variant detection in massively parallel sequencing of individual and pooled samples. *Bioinformatics* **25**(17): 2283-2285.

ANNOVAR – for functional annotation of genetic mutations

Wang K, Li M, Hakonarson H. 2010. ANNOVAR: functional annotation of genetic variants from high-throughput sequencing data. *Nucleic acids research* **38**(16): e164.

Polyphen2 – for prediction of protein consequences of genetic mutations

Adzhubei IA, Schmidt S, Peshkin L, Ramensky VE, Gerasimova A, Bork P, Kondrashov AS, Sunyaev SR. 2010. A method and server for predicting damaging missense mutations. *Nature methods* **7**(4): 248-249.

SIFT (Sorting Intolerant From Tolerant) – for prediction of amino acid changes that affect protein function

[Ng PC](http://www.ncbi.nlm.nih.gov/pubmed?term=Ng PC%5BAuthor%5D&cauthor=true&cauthor_uid=12824425), [Henikoff S](http://www.ncbi.nlm.nih.gov/pubmed?term=Henikoff S%5BAuthor%5D&cauthor=true&cauthor_uid=12824425). 2003. SIFT: Predicting amino acid changes that affect protein function. *Nucleic acids research* **31**(13): 3812-3814.

LRT (Likelihood Ratio Test) – to distinguish deleterious mutations from a large number of variants

S. Chun, J. C. Fay. 2009. Identification of deleterious mutations within three human genomes. *Genome Research***19**(9): 1553-1561.

MutationTaster – for discovery of potential damaging mutations

Schwarz JM, Rödelsperger C, Schuelke M, Seelow D. 2010. MutationTaster evaluates disease-causing potential of sequence alterations. *Nature methods* **7**(8): 575-576.
